# Altered immunometabolic response to fasting in humans living with obesity

**DOI:** 10.1016/j.isci.2025.112872

**Published:** 2025-06-11

**Authors:** Helena Neudorf, Roderick E. Sandilands, Spencer Ursel, Hillary Shaba, Darren Barg, Takeshi Tsusaka, María Dolores Moya-Garzón, Erica Vaz, Patricia Schimweg, Emily L. Goldberg, Jonathan Z. Long, Karsten Krüger, Hashim Islam, Jonathan P. Little

**Affiliations:** 1 School of Health and Exercise Sciences, University of British Columbia Okanagan, Kelowna, BC, Canada; 2 Department of Physiology, University of California, San Francisco, San Francisco, CA, USA; 3 Department of Pathology, Stanford University, Stanford, CA, USA; 4 Sarafan ChEM-H, Stanford University, Stanford, CA, USA; 5 Department of Sports Science, University of Giessen, Giessen, Germany

**Keywords:** immunology, human metabolism

## Abstract

Fasting and ketosis are gaining interest for treating obesity-related immunometabolic dysfunction. We aimed to (1) characterize systemic and T cell immunometabolic responses to a 48-h fast in humans and (2) determine if responses differed between individuals with (O-BMI) and without (L-BMI) obesity (n = 16 per group). Despite similar increases in systemic fat oxidation, increases in blood β-hydroxybutyrate (BHB), BHB-amino acid conjugates, and lysine β-hydroxybutyrylation were blunted in obesity. T cells from the L-BMI group upregulated their relative capacity for fat oxidation while the O-BMI group did not. The O-BMI group had a greater proportion of Th17 cells and secreted more interleukin-17 (IL-17), even after fasting. CD8 expression decreased in both groups and CD4 expression only decreased in the L-BMI group. The balance of anti-to pro-inflammatory cytokines increased less in the O-BMI group. Collectively, these findings show that humans living with obesity have a blunted systemic and T cell immunometabolic response to fasting. NCT05886738.

## Highlights

• Obesity blunts fasting-induced ketogenesis resulting in lower blood β-hydroxybutyrate (BHB)
• Downstream lysine β-hydroxybutyrylation and BHB-amino acid conjugation is lower in obesity
• Obesity impairs T cell metabolic adaptability to fasting
• Obesity negatively impacts the balance of anti-to pro-inflammatory cytokines during fasting

## Graphical abstract

**Figure:**
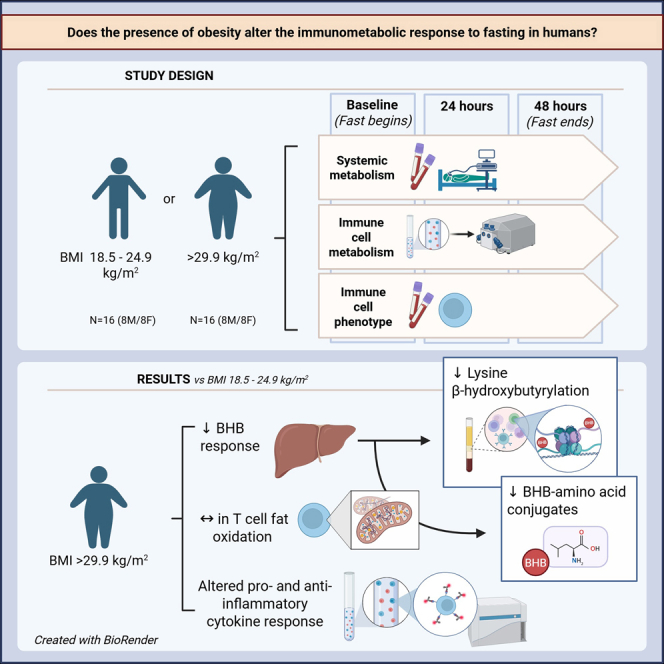

## Introduction

Obesity affects approximately one-sixth of the global adult population^1^ and is associated with a myriad of negative health impacts including dysregulation of the immune system.^2^^,^^3^ Immunological dysregulation contributes to the development of conditions including metabolic syndrome and cardiovascular disease, which often associate with elevated markers of chronic inflammation even in the absence of infection.^4^ Immune cells respond to the microenvironment in obesity by altering their differentiation, phenotype, and functional characteristics. Evidence shows that T cell recruitment to inflamed adipose tissue precedes macrophage recruitment, suggesting that T cell dysfunction is a critical initiating event in the development of obesity-associated inflammation.^5^ Individuals with obesity exhibit a higher ratio of CD8^+^ to CD4^+^ T cells, a larger proportion of more pro-inflammatory (i.e., Th1 and Th17) T cell subsets, decreased immunosuppressive Treg cells,^5^^,^^6^^,^^7^^,^^8^^,^^9^ and elevated pro-inflammatory cytokines such as the chemokine MCP-1.^2^ Importantly, these differences in T cell distribution and inflammatory markers contribute to adipose tissue inflammation and are associated with an increased risk for metabolic disease.^5^^,^^7^^,^^9^ Thus, obesity alters the composition and function of the immune system, particularly T cells, to promote a pro-inflammatory environment.

Although the immunologic consequences of obesity are well-studied, the factors driving these perturbations are less clear—particularly in humans. Obesity is accompanied by altered metabolic homeostasis such as metabolic inflexibility and insulin resistance.^10^^,^^11^ Importantly, metabolites resulting from both systemic and intracellular metabolism can directly influence T cell differentiation and function via receptor-mediated and/or intracellular signaling and epigenetic modifications.^12^^,^^13^ For example, increased GLUT1 expression, mediated by elevated circulating leptin (an adipokine linked with obesity^14^), enhances glycolytic flux and T cell differentiation into inflammatory Th1 and Th17 subsets.^15^^,^^16^ Conversely, low glucose environments or reduced glucose uptake can prevent appropriate T cell activation.^17^ Meanwhile, exposure to excess free fatty acids (FFAs) increases Treg differentiation, decreases pro-inflammatory cytokine secretion via increased β-oxidation,^5^^,^^18^ and Treg cells play a role in maintaining insulin sensitivity.^19^ Therefore, external modification of systemic and thereby cellular metabolism may push T cells toward activation or quiescence. An environment favoring glycolysis appears to polarize T cells toward an activated state, whereas increased fat oxidation encourages suppression of the inflammatory response.^20^

Fasting is an ancient but increasingly popular practice garnering growing research interest as a means to manipulate systemic metabolism. Fasting induces a favorable environment for fat and ketone oxidation, thereby holding potential to improve aspects of metabolic and immune health. For example, fasting can decrease inflammatory cytokine secretion^15^ and improve insulin sensitivity by increased polarization of T cells in mice.^19^ Human studies are more limited, but preliminary evidence suggests that fasting decreases interleukin-6 (IL-6) and MCP-1,^21^ circulating CD4^+^ T cells numbers and activation markers,^21^^,^^22^^,^^23^ and Th2 activation in inflammatory conditions such as asthma.^24^ Additionally, fasting upregulates ketogenesis and growing evidence supports a role for β-hydroxybutyrate (BHB) in mediating some of the immunometabolic changes during fasting. Indeed, research has established a role for BHB as an anti-inflammatory signal resulting in attenuated NLRP3-mediated inflammation, oxidative stress, and endoplasmic reticulum stress.^25^^,^^26^^,^^27^^,^^28^^,^^29^^,^^30^^,^^31^^,^^32^^,^^33^^,^^34^^,^^35^^,^^36^^,^^37^^,^^38^^,^^39^ Additionally, growing evidence suggests that BHB also improves adaptive immune functioning by increasing memory T cell number,^40^^,^^41^ Treg cell number and function, and CD8^+^ T cell oxidative metabolism^40^ and maintaining CD8^+^ T cell function.^40^^,^^42^^,^^43^^,^^44^^,^^45^ Therefore, increasing fat metabolism and ketogenesis by fasting may hold potential to ameliorate some of the immunometabolic perturbations observed in obesity.

Despite this therapeutic potential, it remains unknown if fasting can impact human T cell metabolism thereby altering T cell subsets, phenotype, and functional characteristics. Furthermore, little research has directly compared the immunometabolic response to fasting between individuals living with versus without obesity. The purpose of this study was (1) to characterize systemic and T cell immunometabolic responses to fasting in humans and (2) to determine if responses differed between individuals with a lean body mass index (BMI) compared to those living with obesity. Our findings provide insights into the therapeutic mechanisms by which fasting may alter metabolic responses and impact immune dysregulation in obesity.

## Results

### Anthropometrics

**Figure 1:**
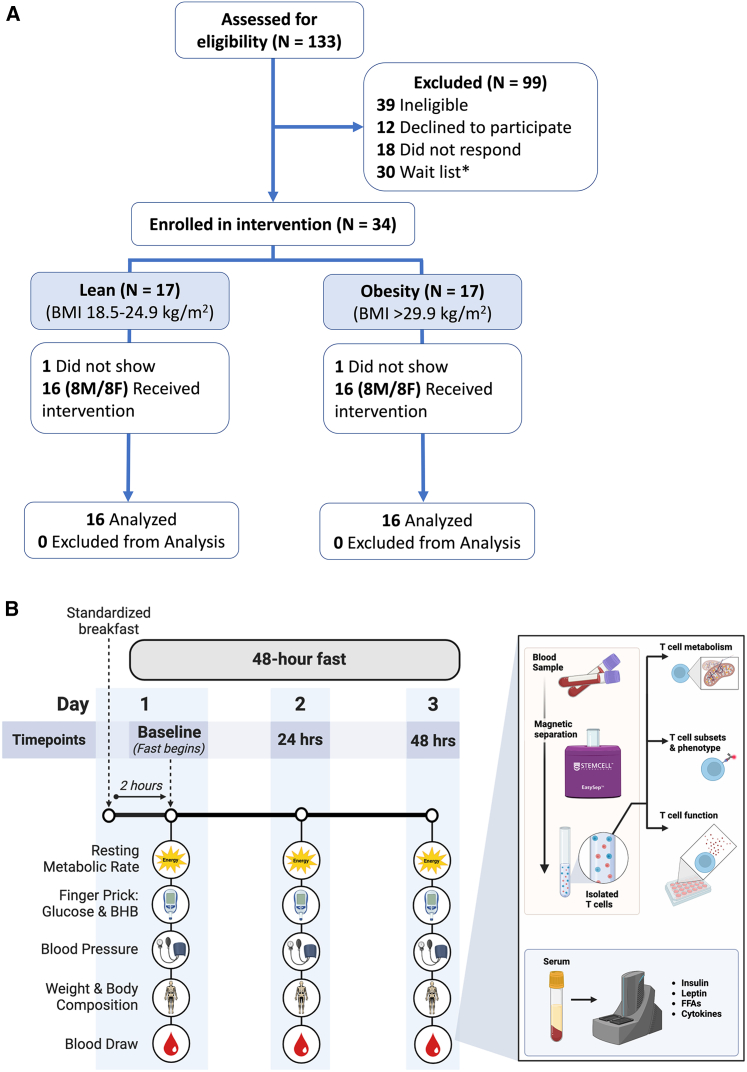
Summary of participant flow and study design (A) CONSORT diagram. ∗Participants were eligible but wait-listed once recruitment had closed but data collection was not yet completed. (B) Schematic depicting the study design across the 48-h fast. Participant baseline measurements were collected 2 h after a standardized breakfast. Participants returned to the lab after 24 and 48 h of fasting and repeated each measurement.

A total of 32 participants completed the fasting intervention (*N* = 8 males and *N* = 8 females per BMI group; Figure 1A). Participant baseline characteristics are reported in Table 1 and are shown disaggregated by sex at baseline in Table S8. As expected, participants in the obese BMI (O-BMI) group had poorer markers of cardiometabolic health at baseline. Despite matching on all key inclusion/exclusion criteria, the O-BMI group was older than the lean BMI (L-BMI) group (38 ± 13 versus 27 ± 11 years). Participants in the O-BMI group experienced greater absolute weight loss compared to the L-BMI group (group × time interaction, *p* < 0.001; Table 1); however, the relative weight loss was approximately 3% of initial body mass in both groups (main effect of time, *p* < 0.001; Table 1). There was no effect of the fast on systolic (main effect of time, *p* = 0.895; Table 1) or diastolic blood pressure (main effect of time, *p* = 0.286; Table 1) in either group. However, resting heart rate increased similarly in both groups across the 48-h fast (main effect of time, *p* < 0.001; Table 1).

**Table 1:** Anthropometrics, vitals, and systemic metabolism during a 48-h fast

	L-BMI			O-BMI			*p* value		
	Baseline	24 h	48 h	Baseline	24 h	48 h	Main effect of time	Main effect of group	Group × time interaction
BMI (kg/m^2^)	22.2 [20.1, 24.3]	21.9 [19.8, 24.0]	21.6 [19.5, 23.7]	35.1 [33.0, 37.2]	34.6 [32.5, 36.7]	34.2 [32.0, 36.3]	∗<0.001	∗<0.001	∗0.001
Body mass (kg)	65.8 [57.2, 74.5]	64.8 [56.2, 73.5]	64.0 [55.3, 72.7]	104.7 [96.0.113.3]	103.0 [94.3, 111.7]	101.7 [93.0.110.3]	∗<0.001	∗<0.001	∗<0.001
% change in body mass	–	−1.66 [−2.15, −1.17]	−2.97 [−3.45, −2.49]	–	−1.54 [−3.45, −2.49]	−2.79 [−3.27, −2.32]	∗<0.001	0.654	0.781
SBP (mmHg)	110 [103, 116]	110 [103, 117]	113 [106, 120]	127 [120, 134]	125 [118, 131]	123 [116, 130]	0.895	∗0.002	0.218
DBP (mmHg)	73 [68, 78]	69 [65, 74]	71 [66, 76]	83 [79, 88]	84 [80, 89]	80 [76, 85]	0.286	∗<0.001	0.135
Pulse (bpm)	67 [60, 74]	70 [63, 77]	78 [71, 85]	68 [62, 75]	68 [62, 75]	73 [66, 80]	∗<0.001	0.726	0.296
REE (kcal/min)	1.25 [1.11, 1.40]	1.24 [1.09, 1.39	1.25 [1.10, 1.40]	1.41 [1.27, 1.55]	1.33 [1.20, 1.47]	1.46 [1.32, 1.60]	0.118	0.113	0.204
Relative REE (kcal/min/kg FFM)	0.024 [0.022, 0.025]	0.023 [0.022, 0.025]	0.024 [0.022, 0.026]	0.022 [0.021, 0.024]	0.023 [0.021, 0.024]	0.025 [0.023, 0.026]	∗0.035	0.751	0.181
RER (a.u.)	0.87 [0.84, 0.89]	0.79 [0.77, 0.82]	0.76 [0.73, 0.78]	0.82 [0.80, 0.85]	0.77 [0.74, 0.79	0.74 [0.72, 0.76]	∗<0.001	∗0.035	0.430
Fat oxidation (mg/min)	60 [45, 76]	97 [81, 113]	108 [92, 123]	86 [72, 101]	110 [95, 124]	138 [123, 152]	∗<0.001	∗0.017	0.208
Relative fat oxidation (mg/min/kg FFM)	1.1 [0.9, 1.4]	1.6 [1.4, 1.9]	2.0 [1.7, 2.3]	1.4 [1.1, 1.7]	1.8 [1.5, 2.1]	2.4 [2.1, 2.6]	∗<0.001	0.086	0.539
Carbohydrate oxidation (mg/min)	165 [133, 198]	80 [46, 115]	57 [25, 89]	144 [114, 174]	72 [42, 102]	39 [8, 69]	∗<0.001	0.370	0.854
Relative carbohydrate oxidation (mg/min/kg FFM)	3.1 [2.6, 3.6]	1.4 [0.9, 2.0]	1.0 [0.5, 1.5]	2.2 [1.8, 2.7]	1.2 [0.7, 1.7]	0.6 [0.1, 1.1]	∗<0.001	0.067	0.301
VO_2_ (mL/min)	259 [228, 289]	261 [230, 292]	264 [234, 295]	293 [265, 322]	281 [252, 310]	310 [281, 339]	0.052	0.096	0.168
Relative VO_2_ (mL/kg FFM/min)	4.85 [4.48, 5.22]	4.90 [4.50, 5.30]	5.04 [4.66, 5.42]	4.67 [4.32, 5.01]	4.62 [4.28, 4.97]	5.25 [4.91, 5.60]	∗0.003	0.692	0.128
VCO_2_ (mL/min)	223 [197, 249]	204 [178, 231]	201 [175, 227]	242 [218, 266]	216 [192, 240]	228 [204, 253]	∗0.002	0.231	0.457
Relative VCO_2_ (mL/kg FFM/min)	0.22 [0.20, 0.25]	0.20 [0.18, 0.23]	0.20 [0.18, 0.23]	0.24 [0.22, 0.27]	0.22 [0.19, 0.24]	0.23 [0.20, 0.25]	∗0.002	0.231	0.457
VE (L/min)	7.29 [6.50, 8.08]	7.36 [6.55, 8.17]	7.56 [6.77, 8.34]	7.83 [7.09, 8.57]	7.28 [6.55, 8.02]	8.06 [7.32, 8.80]	0.055	0.513	0.229

Data are estimated marginal means [95% confidence interval] derived from a linear mixed effects model.

A.u., arbitrary units; BMI, body mass index; DBP, diastolic blood pressure; FFA, free fatty acids; FFM, fat-free mass; REE, resting energy expenditure; RER, respiratory exchange ratio; SBP, systolic blood pressure; VE, ventilation.

### Resting metabolic rate

**Figure 2:**
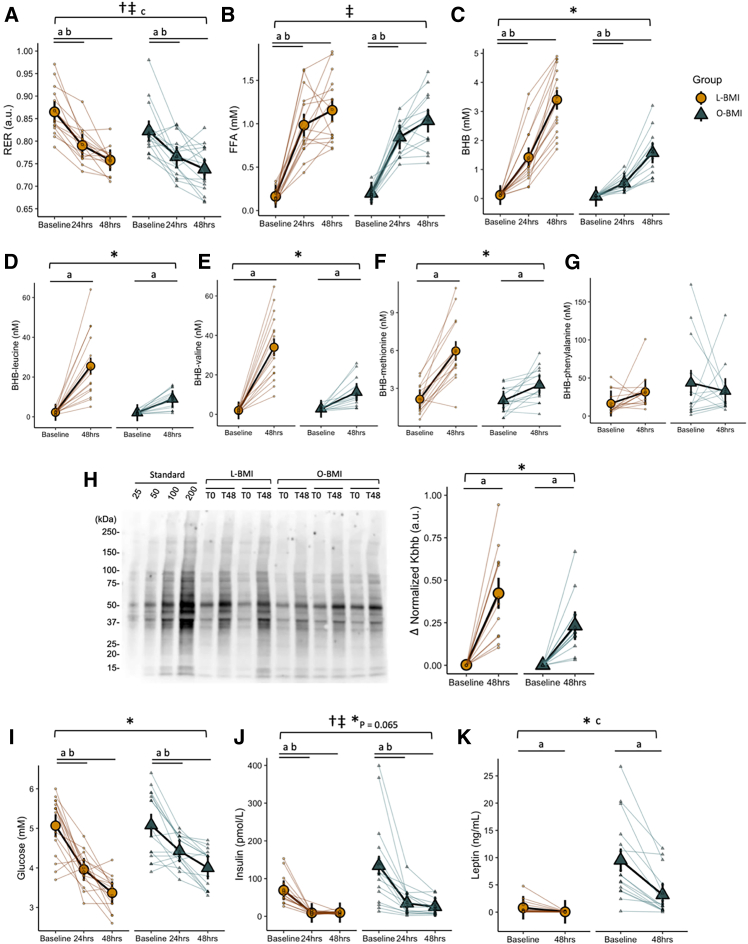
Systemic metabolism and hormones throughout a 48-h fast in individuals with L-BMI and individuals with O-BMI (A) Respiratory exchange ratio (RER) derived by indirect calorimetry. (B) Free fatty acids (FFA) from serum. (C) Capillary beta-hydroxybutyrate (BHB) from finger prick. (E–G) BHB-amino acid conjugates from serum. (H) Representative western blot of beta-hydroxybutyrylated lysine histone residues (Kbhb) from PBMCs and delta of Kbhb normalized to Ponceau stain from PBMCs. (I) Capillary glucose from finger prick. (J) Insulin concentration in serum. (K) Leptin concentration in serum. Large circles or triangles are estimated marginal means and black vertical bars are 95% confidence intervals derived from a linear mixed effects model. Orange circles represent the lean BMI (L-BMI) group and teal triangles represent the obese BMI (O-BMI) group. Small circles or triangles represent individual participant data. *N* = 32; 8 males/8 females per BMI group. ∗Group × time interaction, *p* < 0.05; ‡main effect of time, *p* < 0.05; †main effect of group, *p* < 0.05; ^a^baseline vs. 48 h, *p* < 0.05; ^b^baseline vs. 24 h, *p* < 0.05; ^c^L-BMI vs. O-BMI at baseline, *p* < 0.05.

Systemic metabolism over the 48-h fast was characterized by indirect calorimetry and select blood metabolites. Although the O-BMI group had a lower respiratory exchange ratio (RER) at all time points (main effect of group, *p* = 0.035), RER decreased in response to the fast to a similar extent in both groups (main effect of time, *p* < 0.001; Figure 2A). This corresponded to progressively higher rates of calculated fat oxidation throughout the fast in both groups (main effect of time, *p* < 0.001; Table 1), although the O-BMI group exhibited higher rates of fat oxidation at all time points (main effect of group, *p* = 0.017; Table 1). However, when the rate of fat oxidation was corrected for fat-free mass, the difference between groups was lost. This response was inversely mirrored by declining rates of both absolute and relative carbohydrate oxidation throughout the fast in both groups, as expected (main effect of time, both *p* < 0.001; Table 1). These findings indicate that—despite higher overall fat oxidation in the O-BMI group—the ability to increase whole-body fat oxidation in response to fasting was similar in both L-BMI and O-BMI groups.

### Metabolites and hormones

Serum FFAs increased throughout the fast (main effect of time, *p* < 0.001) to a similar extent in both groups (group × time interaction, *p* = 0.277; Figure 2B). As expected, the concentration of capillary blood BHB increased in both groups throughout the fast. Interestingly, the rise in circulating BHB was blunted in the O-BMI group compared to the L-BMI group such that capillary blood BHB concentration in the O-BMI group was less than half that of the L-BMI group after 48 h of fasting (group × time interaction, *p* < 0.001; Figure 2C). This pattern was replicated in serum BHB-amino acid conjugates BHB-Val, BHB-Leu, and BHB-Met wherein the increase in the O-BMI group was approximately half that of the L-BMI group (all group × time interactions, *p* < 0.001; Figures 2D–2F). BHB-Phe did not follow this pattern and was not significantly different across time in either group (Figure 2G). In line with the blunted increase in circulating BHB and BHB-amino acid conjugates in obesity (Figures 2D–2F), analyses of isolated peripheral blood mononuclear cells (PBMCs) revealed increased β-hydroxybutyrylation of lysine residues with fasting in both groups with the magnitude of β-hydroxybutyrylation in the L-BMI group twice that of the O-BMI (group × time interaction; *p* = 0.031; Figure 2H). These findings indicate an overall blunted ketogenic response to fasting in the O-BMI group, despite similar increases in the availability of circulating FFA for ketogenesis.

Although capillary blood glucose concentration decreased throughout the fast in both groups, the reduction was less in the O-BMI group compared to the L-BMI group (group × time interaction, *p* = 0.029; Figure 2I). Serum insulin decreased by 86% and 81% throughout the fast in the L-BMI and O-BMI groups, respectively (main effect of time, *p* < 0.001; Figure 2J). Despite this, insulin remained significantly higher at all time points in the O-BMI group (main effect of group, *p* = 0.015; Figure 2J). Serum leptin was higher in the O-BMI group at all time points (main effect of group, *p* < 0.001; Figure 2K) but decreased to a greater extent in the O-BMI group after 48 h fasting (group × time interaction, *p* < 0.001; Figure 2K). These findings indicate relative hyperinsulinemia and elevated leptin in the O-BMI group, which was accompanied by lower reduction in blood glucose concentration in response to 48 h of fasting.

### T cell metabolic response

**Figure 3:**
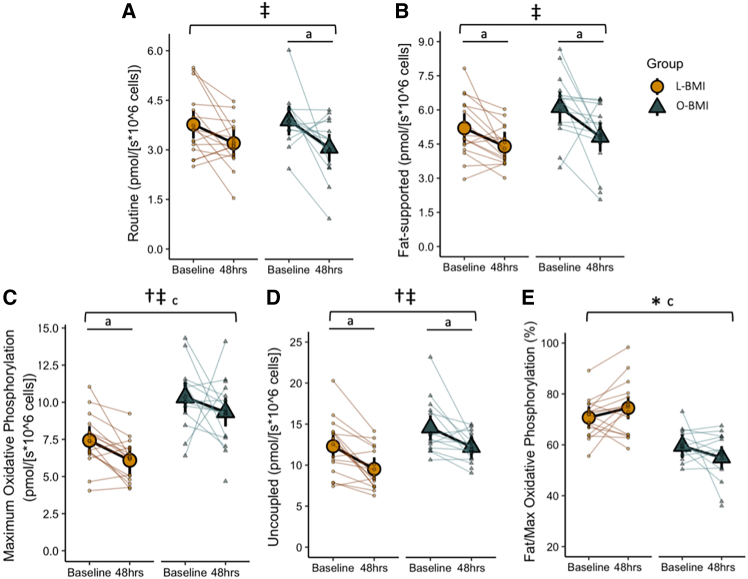
Mitochondrial respiratory states measured in isolated human T cells at baseline and after a 48-h fast in individuals with L-BMI or O-BMI (A) Routine T cell respiration representing respiration in the basal state. (B) T cell respiration due to fat-supported respiration. (C) Maximum oxidative phosphorylation capacity. (D) Uncoupled respiration representing maximum OXPHOS capacity. (E) Proportion of respiration due to fat-supported respiration as a percentage of maximum OXPHOS capacity. Large circles or triangles are estimated marginal means and black vertical bars are 95% confidence intervals derived from a linear mixed effects model. Orange circles represent the lean BMI (L-BMI) group and teal triangles represent the obese BMI (O-BMI) group. Small circles or triangles represent individual participant data. *N* = 32; 8 males/8 females per BMI group. ∗Group × time interaction; ‡main effect of time, *p* < 0.05; †main effect of group, *p* < 0.05; ^a^baseline vs. 48 h, *p* < 0.05; ^c^L-BMI vs. O-BMI at baseline, *p* < 0.05.

To explore if these differential shifts in systemic metabolism resulted in shifts in mitochondrial metabolism at the cellular level, T cells were isolated from participants at baseline and after the 48-h fast and probed for measures of mitochondrial respiration under different substrate and inhibitor conditions. Routine respiration, representing basal respiration fueled by endogenous substrates, decreased to a similar extent in both groups (∼15% in L-BMI and ∼21% in O-BMI) in response to the fast (main effect of time, *p* < 0.001; Figure 3A). T cell fat-supported respiration in the presence of the fatty acid metabolite, octanoylcarnitine, decreased similarly in both groups in response to 48 h of fasting (main effect of time, *p* < 0.001; Figure 3B). Maximum oxidative phosphorylation capacity in the presence of octanoylcarnitine, pyruvate, and succinate was significantly higher in the O-BMI group (main effect of group, *p* < 0.001; Figure 3C) but decreased similarly in both groups (main effect of time, *p* = 0.024; Figure 3C). Uncoupled respiration, representing maximum ET capacity independent of ATP synthase, decreased in both groups following the fast (main effect of time, *p* < 0.001) but was higher at both time points in the O-BMI group (main effect of group, *p* = 0.008; Figure 3D). The proportion of respiration supported by fat was significantly higher in the L-BMI group compared to the O-BMI group, where fat-supported respiration accounted for 70.7 [66.3, 75.1] % of maximal oxidative phosphorylation capacity in the L-BMI group but only 59.5 [54.8, 64.2] % in the O-BMI group (main effect of group, *p* < 0.001; Figure 3E). Furthermore, this percentage increased to 74.5 [70.1, 78.9] % in the L-BMI group but decreased to 55.0 [50.4, 59.5] % in the O-BMI group (group × time interaction, *p* = 0.032; Figure 3E). These results indicate a generalized decrease in multiple T cell mitochondrial respiratory parameters following 48 h of fasting in both groups. Additionally, obesity blunted the ability to upregulate the relative capacity for fat oxidation in response to 48 h of fasting.

### Cell count and phenotypic response

**Figure 4:**
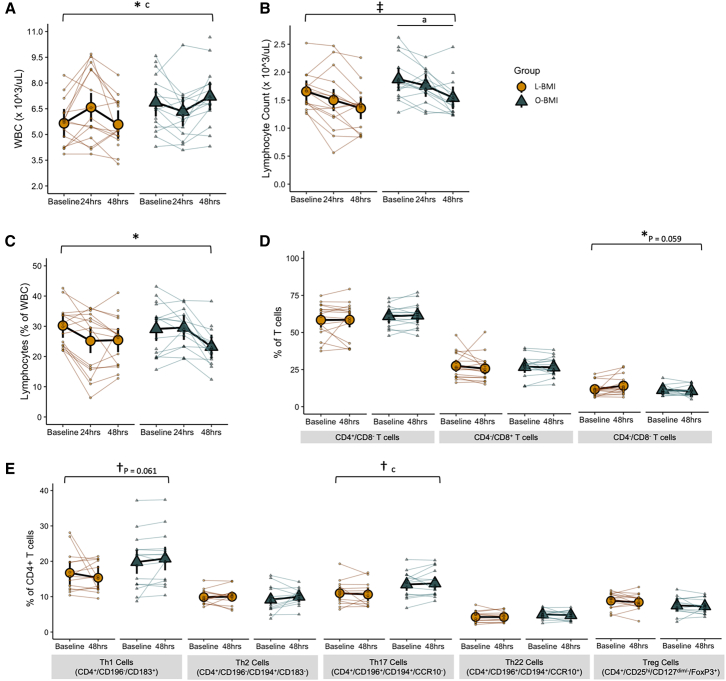
Cell counts and T cell subsets at baseline and after a 48-h fast in individuals with L-BMI or O-BMI (A) Total white blood cell count. (B) Absolute lymphocyte counts in whole blood. (C) Proportion of white bloods cells that were lymphocytes in whole blood. (D) Proportion of isolated T cells derived from whole blood that were CD4^+^/CD8^−^, CD4^−^/CD8^+^, or double-negative (CD4^−^/CD8^−^). (E) Proportion of CD4^+^ T cells that were either Th1, Th2, Th17, Th22, or Treg cells. Large circles or triangles are estimated marginal means and black vertical bars are 95% confidence intervals derived from a linear mixed effects model. Orange circles represent the lean BMI (L-BMI) group and teal triangles represent the obese BMI (O-BMI) group. Small circles or triangles represent individual participant data. *N* = 32; 8 males/8 females per BMI group. ∗Group × time interaction, *p* < 0.05; ‡main effect of time, *p* < 0.05; †main effect of group, *p* < 0.05; ^c^L-BMI vs. O-BMI at baseline, *p* < 0.05.

The potential for fasting-induced shifts in systemic and T cell metabolism to influence cell counts, T cell subsets, and T cell function was characterized by complete blood count and flow cytometric analysis of isolated T cells. Total WBC count was higher in the O-BMI group and also responded in an opposite manner between the two groups (group × time interaction, *p* = 0.002; Figure 4A); WBC count in the L-BMI group peaked at 24 h and then returned to baseline levels after 48 h, while WBC count in the O-BMI group was the lowest at 24 h and peaked at 48 h. Absolute lymphocyte count appeared to be higher in the O-BMI group; however, this difference did not reach statistical significance (main effect of group, *p* = 0.087). However, both groups exhibited a similar reduction in the absolute number of lymphocytes in circulation over the 48-h fast (main effect of time, *p* < 0.001; Figure 4B). Interestingly, while both groups experienced a reduction in the proportion of lymphocytes (% of WBC) in circulation, the timing of this reduction differed by BMI group (group × time interaction, *p* = 0.013; Figure 4C); in the L-BMI group, the lymphocyte percent decreased immediately from baseline to 24 h but then plateaued until 48 h. Meanwhile, the lymphocyte percent in the O-BMI group remained steady from baseline to 24 h but then dropped sharply from 24 h to 48 h. The proportion of isolated pan T cells that were CD4^+^ or CD8^+^ T cells did not change in response to the fast and was not affected by BMI group (Figure 4D). However, the proportion of T cells that were double-negative (CD4^−^/CD8^−^) appeared to be modulated differentially by the fast and BMI group, however this effect did not reach statistical significance (group × time interaction, *p* = 0.059; Figure 4D). The proportions of Th2, Th22, and FoxP3^+^ T regulatory cells were unaffected by either the fast or BMI (Figure 4E). However, the O-BMI group appeared to have a higher proportion of Th1 cells (main effect of group, *p* = 0.061; Figure 4E) and also exhibited a higher proportion of Th17 cells (main effect of group, *p* = 0.023; Figure 4E). These findings support a generally higher number of circulating white blood cells and Th17 cells in obesity, which persist following 48 h fasting.

### T cell function

**Figure 5:**
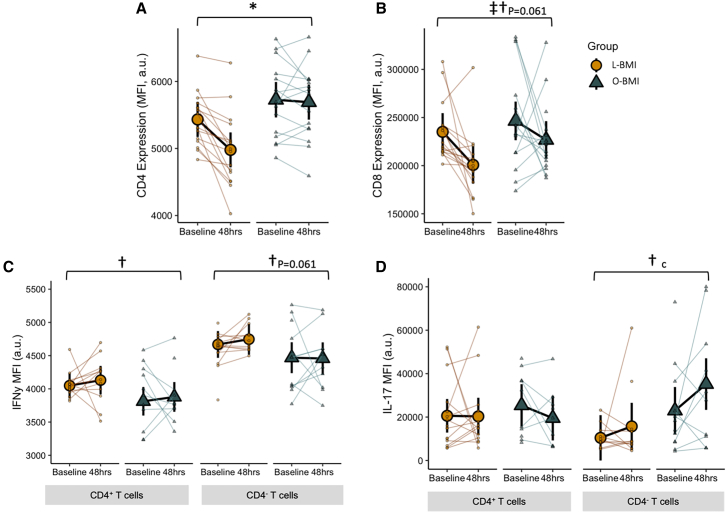
Markers of T cell function before and after a 48-h fast in individuals with L-BMI or O-BMI (A) Expression of CD4 on isolated human T cells. (B) Expression of CD8 on isolated human T cells. (C) Intracellular secretion of IFNγ in isolated human T cells following a 24-h activation culture. (D) Intracellular secretion of IL-17 in isolated human T cells following a 24-h activation culture. Large circles or triangles are estimated marginal means and black vertical bars are 95% confidence intervals derived from a linear mixed effects model. Orange circles represent the lean BMI (L-BMI) group and teal triangles represent the obese BMI (O-BMI) group. Small circles or triangles represent individual participant data. *N* = 32; 8 males/8 females per BMI group. ∗Group × time interaction, *p* < 0.05; ‡main effect of time, *p* < 0.05; †main effect of group, *p* < 0.05; ^c^L-BMI vs. O-BMI at baseline, *p* < 0.05. MFI, median fluorescence intensity.

T cell function was characterized by both receptor expression in isolated T cells in the basal state and by intracellular cytokine abundance following a 24-h activation culture. Despite no effect of the fast or BMI on the proportion of CD4^+^ T cells (Figure 4D), CD4 receptor expression dropped by 8% in response to the fast in the L-BMI group but remained unaffected in the O-BMI group (group × time interaction, *p* = 0.004; Figure 5A). CD8 expression decreased by 15% and 8% in the L-BMI and O-BMI groups, respectively, in response to the fast (main effect of time, *p* = 0.008; main effect of group, *p* = 0.061; Figure 5B).

To interrogate the T cell response to an activating stimulus (CD3/CD28), T cells were cultured for 24 h and intracellular cytokine production was characterized by flow cytometry in CD4^+^ and CD4^−^ cells (presumed to be cytotoxic CD8^+^ T cells; Figures 5C–5E). Interestingly, interferon gamma (IFNγ) secretion from CD4^+^ T cells was higher in the L-BMI group (main effect of group, *p* = 0.035; Figure 5C), and appeared to be higher from CD4^−^ T cells from the L-BMI group as well, although this did not reach statistical significance (main effect of group; *p* = 0.061; Figure 5C). Despite the differential effect of BMI, IFNγ was not affected by the fast. Intracellular IL-17 secretion from CD4^+^ T cells was unaffected by both BMI and the fast (Figure 5D). However, IL-17 secretion from CD4^−^ T cells was higher in the O-BMI group. Collectively, these findings show a blunted decrease in CD4 receptor expression in response to fasting in the O-BMI group compared to their lean counterparts. Although reduced CD4 expression was not accompanied by fasting-induced alterations in T cell cytokine secretion, the differential T cell IFN-γ (higher in L-BMI) and IL-17 (higher in O-BMI) secretion in the basal state are indicative of altered T cell functionality in obesity.

### Circulating cytokines

**Figure 6:**
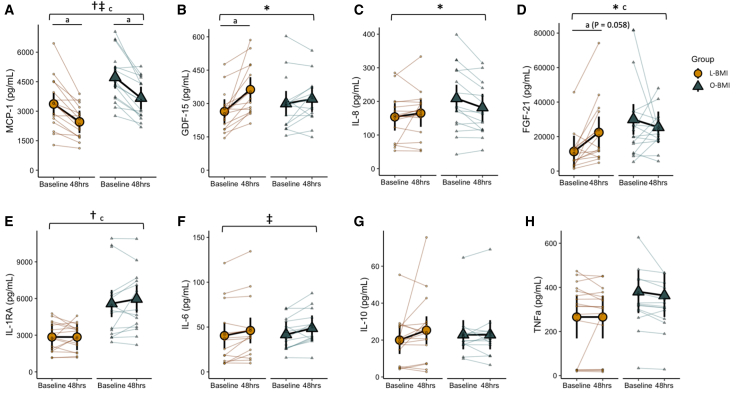
Plasma cytokines before and after a 48-h fast in individuals with L-BMI or O-BMI. Plasma cytokine responses (A) Monocyte chemoattractant protein (MCP)-1. (B) Growth/differentiation factor (GDF)-15. (C) Interleukin (IL)-8. (D) Fibroblast growth factor (FGF)-21. (E) Interleukin (IL)-1 receptor antagonist (RA). (F) Interleukin (IL)-6. (G) Interleukin (IL)-10. (H) Tumor necrosis factor (TNF)-alpha. Large circles or triangles are estimated marginal means and black vertical bars are 95% confidence intervals derived from a linear mixed effects model. Orange circles represent the lean BMI (L-BMI) group and teal triangles represent the obese BMI (O-BMI) group. Small circles or triangles represent individual participant data. *N* = 32; 8 males/8 females per BMI group. ∗Group × time interaction, *p* < 0.05; ‡main effect of time, *p* < 0.05; †main effect of group, *p* < 0.05; ^a^baseline vs. 48 h, *p* < 0.05; ^c^L-BMI vs. O-BMI at baseline, *p* < 0.05. MFI, median fluorescence intensity.

To quantify markers of systemic inflammation, a panel of circulating serum cytokines was measured before and after 48 h of fasting. MCP-1 decreased by 27% and 22% in the L-BMI and O-BMI groups, respectively, in response to the fast (main effect of time, *p* < 0.001) but was higher in the O-BMI group at both time points (main effect of group, *p* = 0.002; Figure 6A). Growth/differentiation factor (GDF)-15 increased 38% in the L-BMI group after the fast but was unchanged in the O-BMI group (Group × Time interaction, *p* = 0.015; Figure 6B). Conversely, IL-8, which was lower in the L-BMI group at baseline, increased 7% in the L-BMI group but decreased 13% in the O-BMI group in response to the fast (group × time interaction, *p* = 0.007; Figure 6C). Fibroblast growth factor (FGF)-21 was much lower at baseline in the L-BMI group and increased 97% in response to the fast, while the O-BMI group, which started higher at baseline, experienced a reduction of 15% (group × time interaction, *p* = 0.030; Figure 6D). IL-1RA was unchanged in response to the fast but was significantly higher in the O-BMI group compared to the L-BMI group at both time points (main effect of group, *p* = 0.001; Figure 6E). IL-6 increased by 14% and 17% in the L-BMI and O-BMI groups, respectively, in response to the fast (main effect of time, *p* < 0.001; Figure 6F). IL-10 and tumor necrosis factor alpha (TNFα) were unchanged in response to the fast and were not significantly different between groups (Figures 6G and 6H). These plasma cytokine data support a generally heightened basal inflammatory state in obesity coupled with altered GDF-15 and FGF-21 responses to 48 h fasting.

## Discussion

This study aimed to determine the impact of a 48 h fast on systemic metabolism, T cell respiration, and T cell functional characteristics and whether responses were altered by the presence of obesity. As expected, a 48 h fast dramatically increased systemic fat metabolism as shown by decreased RER (Figure 1A) and increased fat oxidation (Table 1). While the absolute rate of fat oxidation was higher in the O-BMI group, the effect of BMI was lost when corrected for fat-free mass. Additionally, the absolute rate of carbohydrate oxidation decreased in both groups similarly whereas absolute resting energy expenditure was unaffected by the fast (Table 1). Consistent with previous reports,^46^^,^^47^^,^^48^ obesity blunted the ketogenic response to fasting (Figure 2C). Intriguingly, we report here, that the blunted increase in ketosis in humans with obesity is also accompanied by a lower rise in serum ketone metabolites (BHB-Val, BHB-Leu, and BHB-Met) along with a lower increase in PBMC lysine β-hydroxybutyrylation. Given that BHB-amino acid metabolites are produced by enzymatic conjugation in cells that express the enzyme CNDP2^49^ and that lysine β-hydroxybutyrylation is a post-translational modification of proteins regulated by mass action by class I histone deacetylases (HDACs),^50^ these findings indicate functional cellular consequences of the reduced rise in blood BHB in response to fasting in individuals living with obesity. While it is unknown which mechanisms drive this differential ketogenic response, the O-BMI group exhibited relative hyperinsulinemia at all time points compared to the L-BMI group (Figure 2I). Lipolysis, a requisite for ketogenesis, is negatively regulated by insulin, yet the concentration of FFAs in circulation was comparable between groups (Figure 2B). Additionally, insulin is a negative regulator of both the transcription and flux of the rate-limiting ketogenic enzyme,^51^ HMGCS2, such that despite similar availability of FFAs, ketogenic potential is likely lower in the setting of (relative) hyperinsulinemia. The reduced impact of 48 h of fasting on lowering blood glucose in individuals living with obesity also supports an impact of hyperinsulinemia (and/or insulin resistance) on blunting metabolic responses to fasting in the context of obesity. Collectively, the systemic metabolism data indicates that the presence of obesity blunted the metabolic response to fasting, including reducing functional ketone-related signaling at the cellular level.

Interestingly, the responses in systemic metabolism to fasting were partially reflected in our detailed analyses of T cell mitochondrial respiration. Routine (basal) respiration decreased similarly in both groups in response to the fast, indicating reduced flux through mitochondrial ATP-producing metabolic pathways in response to nutrient deprivation (Figure 3A). This is in agreement with previous findings that fasting decreased mitochondrial activity and capacity in skeletal muscle and liver mitochondria across species.^52^^,^^53^^,^^54^ While both the L-BMI and O-BMI groups experienced a reduction in all respiratory states with fasting (likely to account for an overall decreased energy availability), the O-BMI group exhibited a slightly higher maximal capacity for oxidative phosphorylation at both time points (Figure 3C). However, fat-supported respiration as expressed as a % of maximal oxidative phosphorylation capacity was higher in L-BMI group at baseline and in response to the 48-h fast (Figure 3E). Taken together, T cells respond to fasting by downregulating their mitochondrial respiration rate regardless of BMI. However, T cells from individuals living with obesity are unable to upregulate their relative capacity for fat oxidation and therefore exhibit a blunted response to fasting not only systemically but also at the cellular level.

The inability of T cells from individuals with obesity to upregulate the capacity for fat oxidation upon fasting is metabolically expensive and maladaptive in an environment of negative energy balance and progressively limited glucose availability. Upon T cell activation, the metabolic rate rises due to increases in both aerobic glycolysis and oxidative phosphorylation.^13^^,^^55^ Additionally, it has been observed that T cells exhibit higher constitutive activation in an obese microenvironment^56^ and these activated cells prefer glucose oxidation.^57^ Although speculative, this phenomenon may explain the higher rates of respiration and reduced relative capacity for fat versus carbohydrate oxidation observed in the O-BMI group. Supporting this, we observed that the O-BMI group had a greater proportion of the pro-inflammatory Th1 and Th17 subsets in circulation, and fasting was insufficient to shift this balance back toward that of the L-BMI group (Figure 4E). Furthermore, T cells from the O-BMI group secreted more of the highly inflammatory cytokine, IL-17, compared to the L-BMI group. This difference was only detectable in Tc17 cells (Figure 5D) which are also known to secrete IL-17.^58^ IL-17 facilitates crosstalk between the adaptive and innate immune systems by promoting neutrophil activation and is a key player in the pathogenesis of inflammatory disease.^59^ These findings reflect differences previously observed in adipose tissue, wherein the obese microenvironment polarizes T cells toward the proinflammatory subsets, Th1 and Th17, and away from subsets which function to regulate and suppress the immune response such as Treg cells.^5^^,^^6^^,^^7^^,^^8^^,^^9^ CD4 receptor expression, a co-receptor required for T cell activation, decreased in T cells from the L-BMI group with fasting but not in the O-BMI group, suggesting that immune responsiveness of CD4^+^ T cells was unable to be suppressed in this group. Although the influence of T cell activation on T cell metabolism is bi-directional, the higher proportions of circulating pro-inflammatory T cells and their lower threshold to activation observed in the O-BMI group may explain these cells’ inability to adapt to the energetic stress of fasting by increasing capacity for fat-supported respiration and instead maintained a metabolically inefficient phenotype.

In addition to intracellular metabolism, metabolites present in circulation also influence the polarization of T cells. Indeed, it has been widely shown that the ketone body BHB increases histone acetylation,^34^^,^^45^^,^^60^ and in T cells this increases anti-viral IFN-γ secretion upon activation and enhances CD8^+^ cytotoxic T cell responses.^45^ Interestingly, we observed greater anti-CD3/CD28-stimulated IFN-γ secretion in the L-BMI group compared to the O-BMI group (Figure 5C). Notably, the magnitude of IFN-γ secretion was not suppressed with fasting despite decreased T cell mitochondrial respiration and downregulated CD4 and CD8 receptor expression (Figures 3, 5A, and 5B). This preservation of responsiveness may be explained by recent research that has established a role for BHB to act as an alternative fuel source in T cells when BHB concentrations meet or exceed 2 mM,^42^^,^^45^ thereby maintaining T cell functionality in the presence of low glucose availability.^42^ Even in the presence of normal (5 mM) glucose availability, 2 mM BHB provides substrate for approximately 50% of the acetyl CoA pool.^45^ Meanwhile, knock out of the ketolytic enzyme, BDH1, does not affect the size of the acetyl CoA pool but forces T cells to derive their acetyl CoA primarily from acetate and glucose under 2 mM BHB and 5 mM glucose conditions.^45^ Although we were not able to quantify *in vivo* oxidation of BHB by circulating T cells, serum BHB reached a mean of 3.7 mM after the 48-h fast in the L-BMI group but only 1.9 mM in the O-BMI group, such that the O-BMI group may not have passed this critical concentration threshold necessary to induce significant BHB oxidation and switch metabolic preference away from glucose-derived sources, as suggested by their inability to upregulate relative fat-supported respiration (Figure 3E).

In addition to its function as a histone deacetylase inhibitor, BHB has also been found to leave its own post-translational modification on proteins, lysine β-hydroxybutyrylation (Kbhb).^61^^,^^62^^,^^63^ The magnitude of this modification appears to be highly related to a cell’s exposure to BHB concentration, with specific lysine β-hydroxybutyrylation on histones linked to increased expression of genes involved with regulating catabolism of amino acids, redox balance, PPAR signaling and fat oxidation, insulin signaling, and oxidative phosphorylation.^61^ Lysine β-hydroxybutyrylation also occurs on non-histone proteins^50^ as supported by our data showing broad increases across proteins of different molecular weights in human PBMCs after 48 h fasting. Interestingly, lower levels of Kbhb following the fast were seen in the O-BMI group (Figures 5E and 5F) coinciding with lower circulating concentration of BHB and supporting an *in vivo* functional relevance of the blunted ketogenic response to fasting in human immune cells. To our knowledge, this is the first study demonstrating that Kbhb occurs in isolated human cells following an *in vivo* ketogenic intervention. Therefore, it will be interesting to further probe whether the blunted rise in BHB in individuals with obesity might be mechanistically linked to T cell mitochondrial and functional responses to fasting.

### Conclusion

In conclusion, the presence of obesity blunted the adaptive ketogenic response to 48 h of fasting, likely explained at least in part by higher insulin relative to the L-BMI group. This blunted systemic ketogenic response extended to functional cellular endpoints, including a lower increase in secondary BHB-amino acid conjugate metabolites, reduced intracellular lysine β-hydroxybutyrylation, and a failure of circulating T cells to upregulate relative fat-supported respiration in individuals living with obesity. This lowered metabolic adaptation coincided with a resistance to downregulating T cell responsiveness. Future research should further probe these differential immunometabolic responses in obesity and the potential consequences for the therapeutic application of fasting in metabolic disease.

### Limitations of the study

The present study is among the first to directly compare T cell mitochondrial respiratory function in response to fasting in humans with differing BMIs and examine effects on systemic and cellular markers of inflammation. Although intriguing, these findings are limited to T cells present in circulation as it was not feasible in humans to capture immunometabolic responses of T cells residing within key metabolic tissues (e.g., adipose or liver) where important disease-modifying effects are likely more pronounced. Nevertheless, effector T cells cycle between lymphatic vessels, secondary lymphoid organs, circulation, and tissues such that the T cells characterized in circulation are likely to reflect the T cells present across tissues.^64^ Additionally, we quantified the metabolism of pan-T cells rather than specific subsets because it was not feasible to isolate a sufficient number of T cells given the large blood collection volume that would have been required for accurate mitochondrial profiling of T cell subsets by high-resolution respirometry. It is possible that there are subset-specific metabolic responses to fasting or subset differences between the L-BMI and O-BMI groups. However, the proportions and therefore the contribution of each subset within groups remained stable throughout the fast such that the composition of the pan-T cells being interrogated for metabolic phenotype was unlikely to explain the differences observed. Nevertheless, future research should explore metabolism in the different T cell subsets. Despite matching on all key inclusion/exclusion criteria, the O-BMI group was older (∼38 vs. 27 years) in this study, owing to lower recruitment response rates from younger individuals living with obesity. Although we cannot completely rule out the combination of obesity and slightly older age may have contributed to the findings, both groups were matched for physical activity, and did not have a diagnosis for metabolic disease, inflammatory disease, or cancer, and were not receiving medical treatments known to influence these parameters. Additionally, previous research has reported a blunting effect of obesity on the circulating ketone bodies following fasting.^65^ Therefore, we feel that it was very likely the presence of obesity, and not the age difference between the groups, were driving the responses seen.

## Resource availability

### Lead contact

Further information and requests for resources and reagents should be directed to and will be fulfilled by the lead contact, Dr. Jonathan Little (jonathan.little@ubc.ca).

### Materials availability

All materials used in this study are listed in the STAR Methods and are available for purchase from the manufacturer. This study did not generate new unique reagents.

### Data and code availability

• Data collected for this study, including individual deidentified participant data and a data dictionary defining each field in the set, will be made available to others with an academic affiliation upon reasonable request to the lead contact, subject to their approval.
• All original R code used for data analysis will be made available to others with an academic affiliation upon reasonable request to the lead contact, subject to their approval.
• Any additional information required to reanalyze the data reported in this paper will be made available to others with an academic affiliation upon reasonable request to the lead contact, subject to their approval.

## Acknowledgments

The authors would like to thank the dedicated study participants for volunteering their time and effort and for their interest in this study, as this study would not be possible without them. The authors would also like to thank Mr. Garett Jackson for his assistance with blood draws. This study was funded by an 10.13039/501100000038NSERC Discovery Grant (RGPIN-2019-05204) awarded to J.P.L.

## Author contributions

H.N., H.I., and J.P.L. conceived and designed the study and had unrestricted access to the data. H.N., R.S., H.S., E.V., and H.I. conducted the in-person experiments and collected participant samples. H.N., R.S., S.U., H.S., D.B., T.T., M.D.M.-G., P.S., E.V., and H.I. acquired the data. H.N. performed statistical analyses and wrote the manuscript with assistance and oversight from H.I. and J.P.L. J.P.L. secured funding for the trial. All authors contributed to revising the figures and manuscript. All authors approved the final version of this manuscript and take full responsibility for its content including the accuracy of the data and fidelity to the protocol.

## Declaration of interests

J.P.L. is Chief Scientific Officer for the not-for-profit Institute for Personalized Therapeutic Nutrition. J.P.L. holds founder shares in Metabolic Insights Inc., a for-profit company that developed non-invasive metabolic monitoring devices.

## STAR★Methods

### Key resources table

**Table:** 

REAGENT or RESOURCE	SOURCE	IDENTIFIER
**Antibodies**		

CD45 VioBlue	Miltenyi Biotec	Cat.No.130-110-637
CD4 VioGreen	Miltenyi Biotec	Cat.No.130-113-230
CD4 FITC	Miltenyi Biotec	Cat.No.130-114-531
CD25 BioBright B515	Miltenyi Biotec	Cat.No.130-113-287
CD127 PE	Miltenyi Biotec	Cat.No.130-133-753
CD8 PerCP-Vio700	Miltenyi Biotec	Cat.No.130-110-682
FoxP3 VioR667	Miltenyi Biotec	Cat.No.130-122-994
Tandem Signal Enhancer	Miltenyi Biotec	Cat.No.130-099-887
CD183 VioBright FITC	Miltenyi Biotec	Cat.No.130-118-545
CD194 PE-Vio770	Miltenyi Biotec	Cat.No.130-118-359
CD196 PE-Vio615	Miltenyi Biotec	Cat.No.130-120-459
CCR10 APC	Miltenyi Biotec	Cat.No.130-120-406
CCR10 PE	Miltenyi Biotec	Cat.No.130-120-407
IFNy VioBlue	Miltenyi Biotec	Cat.No.130-119-577
IL-4 APC	Miltenyi Biotec	Cat.No.130-114-843
IL-17	Miltenyi Biotec	Cat.No.130-118-249
7-AAD	Miltenyi Biotec	Cat.No.130-111-568
Viobility™ 405/520 Fixable Dye	Miltenyi Biotec	Cat.No.130-130-404
CD3 Monoclonal Antibody (OKT3), Functional Grade, eBioscience™	Invitrogen	Cat.No.16-0037-81
CD28 Monoclonal Antibody (JJ319), Functional Grade, eBioscience™	Invitrogen	Cat.No.16-0289
β-Hydroxybutyryllysine (Kbhb)	PTM BIO	Cat.No. PTM-1201RM
Goat anti-Rabbit IgG HRP	Thermo Scientific	Cat.No. 31460

**Biological samples**		

Human whole blood	Exercise Metabolism and Inflammation Laboratory at the University of British Columbia, Okanagan Campus, Canada	
Human serum	Exercise Metabolism and Inflammation Laboratory at the University of British Columbia, Okanagan Campus, Canada	
Human T cells (isolated from human whole blood)	Exercise Metabolism and Inflammation Laboratory at the University of British Columbia, Okanagan Campus, Canada	
Human PBMCs (isolated from human whole blood)	Exercise Metabolism and Inflammation Laboratory at the University of British Columbia, Okanagan Campus, Canada	

**Chemicals, peptides, and recombinant proteins**		

Fixation Buffer	Miltenyi Biotec	Cat.No.130-122-981
Permeabilization Buffer	Miltenyi Biotec	Cat.No.130-122-981
eBioscience™ IC Fixation Buffer	Invitrogen	Cat.No.00-8222-49
eBioscience™ Permeabilization Buffer (10X)	Invitrogen	Cat.No.00-8333-56
RPMI 1640		
eBioscience™ Brefeldin A Solution (1000X)	Invitrogen	Cat.No.00-4506-51
D-(+)-Glucose solution	Sigma-Aldrich	G8769
Leucosep^TM^ Tubes	Greiner Bio-One	163288
Histopaque	Sigma-Aldrich	10771
Digitonin	Fluka	37008
Malate	Sigma-Aldrich	M1000
DL-Octanoyl-carnitine-HCl, C15H30NO4Cl	TOCRIS Bioscience	No. 0605
Adenosine 5′diphosphate (ADP)	Cal-Biochem	117105
Pyruvate	Sigma-Aldrich	P2256
Cytochrome *c*	Sigma-Aldrich	C7752
Succinate	Sigma-Aldrich	S2378
Carbonyl cyanide *m*-chlorophenyl hydrazone (CCCP)	Sigma-Aldrich	C2759
Antimycin A	Sigma-Aldrich	A8674
Mitochondrial Respiration Buffer MiR05 Buffer	Oroboros	60101–01
RIPA buffer	Thermo Scientific	89901
Protease inhibitor cocktail	Thermo Scientific	A32965
4x Laemmli buffer	Bio-Rad	1610747
β-mercaptoethanol	Sigma-Aldrich	97622
SuperSignal™ West Pico PLUS Chemiluminescent Substrate	Thermo Scientific	34577
BHB-Phe (N-β-hydroxybutyryl-phenylalanine)	doi: https://doi.org/10.1101/2024.09.09.612087	
BHB-Val (N-β-hydroxybutyryl-valine)	doi: https://doi.org/10.1101/2024.09.09.612087	
BHB-Leu (N-β-hydroxybutyryl-leucine)	doi: https://doi.org/10.1101/2024.09.09.612087	
BHB-Met (N-β-hydroxybutyryl-methionine)	doi: https://doi.org/10.1101/2024.09.09.612087	
Ammonium acetate	Sigma	A2706
Ammonium hydroxide	Fisher	A669-500

**Critical commercial assays**		

Human Insulin ELISA Kit	Cayman Chemical	Cat.No.90095
Wako NEFA-HR(2) Assay	FujiFilm	
EasySep Direct Human T cell Isolation Kit	StemCell Technologies	Cat.No.19661
R-PLEX Human GDF-15 Assay	MesoScale Discovery	Cat.No.K151YDR
U-PLEX Custom Metabolic Group 1 (human) Assays	MesoScale Discovery	Cat.No.K151ACM

**Software and algorithms**		

R Studio, Version 4.0.3 (Packages: sjPlot; lme4; lmerTest; emmeans)		https://posit.co/download/rstudio-desktop/#download

**Other**		

Ensure Plus Calories	Abbott	
FreeStyle Precision β-ketone Test Strips	Abbott	
FreeStyle Precision Blood Glucose Test Strips	Abbott	
FreeStyle Precision *Neo* Blood Glucose Monitoring System	Abbott	
BD Vacutainer® EDTA Tubes	BD	SKU366643; SKU367863
BD Vacutainer® SST^TM^ Tubes	BD	SKU367986
TrueOne® 2400	Parvo Medics	
DxH 500 Hematology Analyzer	Beckman Coulter	
CytoFlex S	Beckman Coulter	
NH_2_ 100 Å LC column	Phenomenex	00B-4378-E0

### Experimental model and study participant details

The present paper reports outcomes resulting from a two-group pre-post experimental trial which aimed to determine how 48 h of fasting impacted the metabolism, subsets, and function of T cells and if these responses differed between T cells derived from lean individuals versus individuals with obesity. This trial was approved by the University of British Columbia Clinical Research Ethics Board (H22-03605) and was registered with ClinicalTrials.gov (NCT05886738). All participants provided written informed consent prior to participation and no adverse events were reported.

#### Participants

Participants were recruited between July 2023 and July 2024 from the Central Okanagan region in British Columbia, Canada. Participants were recruited by word-of-mouth, posters around UBC and the community, online advertisements, and email invitations to participants from past studies who had agreed to be contacted for future research. Recruitment was closed once 8 male and 8 female participants in each BMI group (*N* = 16 total per BMI group) had received the intervention (CONSORT diagram, Figure 1A). Although our study was not specifically designed for sex-based analyses, equal numbers of males and females were enrolled in each BMI group, and all data are reported disaggregated by sex in Tables S8–S12. Interested volunteers were screened via completion of a standardized screening questionnaire and physical activity was assessed via completion of the Canadian Society for Exercise Physiology (CSEP) Get Active Questionnaire (GAQ). Interested volunteers were included if they had a BMI either between 18.5 and 24.9 kg/m^2^ (lean BMI group, L-BMI) or ≥30 kg/m^2^ (group living with obesity, O-BMI). The main inclusion criteria were 19–69 years of age and physically inactive (defined as accumulating <150 min of moderate-to-vigorous physical activity per week or participating in moderate-to-vigorous physical activity on less than three days per week based on the current Canadian physical activity guidelines^66^). Additionally, all participants had to be able to read and understand English in order for written informed consent to be collected and to understand the study instructions. Interested volunteers were excluded if they had a diagnosed autoimmune or inflammatory disease, had a cancer diagnosis and/or treatment within the last five years, a type 1 or 2 diabetes diagnosis, a history of cardiovascular events (e.g., heart attack, stroke), and/or were currently pregnant. Additionally, interested volunteers were excluded if they took glucose-lowering or thyroid medications, or any other medication with known effects on glucose or lipid metabolism, or if they currently smoked cigarettes or could not refrain from smoking/using cannabis for the duration of the study. Finally, individuals that actively took ketone supplements, practiced intermittent fasting with regular periods of fasting ≥24 h, followed a ketogenic diet, or were actively losing or gaining weight (>4 kg weight loss or gain in last month) were excluded from the study.

#### Study visit procedures

All testing visits were conducted at the University of British Columbia, Okanagan Campus, Canada. Participants attended three testing visits across three days during which measures were collected at Baseline (Day 1), after 24 h of fasting (Day 2), and after 48 h of fasting (Day 3). The study design is depicted in Figure 1B.

##### Study visit 1

Participants arrived to the lab for Baseline pre-testing in the overnight-fasted (≥10 h) state and having avoided moderate-to-vigorous physical activity and alcohol the day prior. Upon arrival, the study procedures were reviewed with the participant, written informed consent was obtained, and anthropometrics (height, body mass, BMI, and body composition) were collected. Participants then consumed a standardized meal replacement drink containing 11 g of fat, 50 g of carbohydrates, and 13 g of protein (Ensure Plus Calories, Abbott; full macronutrient composition listed in Table S1) for breakfast. In order to begin the fast in the post-absorptive but not fasted state, Baseline measures were collected after 2 h of quiet sitting. This 2-h postprandial timepoint was considered their Baseline and represented the beginning of the 48-h fast. Baseline resting metabolic rate (RMR) was collected for 20 min, following which blood pressure was measured, and capillary blood glucose and capillary blood BHB were measured by finger prick. Venous blood was also collected into EDTA and Serum Separator tubes (BD Vacutainer) by standard venipuncture from an antecubital vein.

##### Study visit 2

Participants returned to the laboratory on the second day, 24 h following Baseline blood sample on the first day. Anthropometrics, RMR, blood pressure, and capillary blood glucose and BHB were collected in the same manner as the first testing visit. Venous blood was again collected into EDTA and Serum Separator tubes (BD Vacutainer).

##### Study visit 3

Participants returned to the laboratory on the third and final day, 48 h following their Baseline blood sampling timepoint on the first day. Anthropometrics, RMR, blood pressure, and capillary blood glucose and BHB were collected again, and venous blood was collected into EDTA and Serum Separator tubes (BD Vacutainer). This was considered their “post” 48-h fasting timepoint.

### Method details

#### Resting metabolic rate

RMR was quantified by indirect calorimetry at every timepoint. Participants were fitted with a mask and then rested in the supine position for 20 min. The expired air was analyzed by a metabolic cart (TrueOne 2400, Parvo Medics) for the following parameters: respiratory exchange ratio (RER), VO_2_ (L/min), VCO_2_ (L/min), ventilation (VE, L/min), resting energy expenditure (REE, kcal/min), and rates of carbohydrate and fat oxidation (g/min).

#### Immunological outcomes

##### Complete blood count

Fresh EDTA blood was immediately analyzed by hematology analyzer (DxH 500, Beckman Coulter) following each blood draw (Baseline, 24 h, 48 h) to obtain a 21-part blood differential. Only white blood cell (WBC) count, lymphocyte count, and lymphocyte percent (% of WBC) are reported in the present paper.

##### T cell isolation

Pan T cells were isolated at Baseline and following 48 h of fasting. Pan T cells were immediately isolated from fresh EDTA blood using the EasySep Direct Human T cell Isolation Kit (StemCell Technologies; Cat. No. 19661) according to the manufacturer’s directions. Following isolation, T cells were washed in PBS and centrifuged for 10 min at 300 g at room temperature. The isolated T cells were then counted and used for all of the following assays.

##### T cell respiration

T cell bioenergetics were characterized by quantitative high-resolution respirometry (Oxygraph 2K, Oroboros Instruments) in freshly isolated, unstimulated pan T cells in duplicate at Baseline and after participants had fasted for 48 h. The substrate-uncoupler-inhibitor titration (SUIT) protocol is depicted in Figure S1. To quantify Routine respiration, 2 × 10^6^ cells (ce) resuspended in mitochondrial respiration buffer (MiR05) were added to each 0.5 mL chamber. Next, cells were permeabilized with digitonin (Dig; 5 μg/mL final concentration) prior to addition of malate (M; 2 mM) and octanoylcarnitine (Oct; 10 μM). Fat-supported oxidative phosphorylation (FAO) was stimulated via addition of ADP (D; 2.5 mM). Next, pyruvate (P; 5 mM) was added to stimulate carbohydrate-supported respiration through complex (C)I, followed by cytochrome *c* (c; 10 μM) to determine mitochondrial outer membrane integrity. Succinate (S; 10 mM) was added to achieve maximal oxidative phosphorylation capacity through CI and CII. Carbonyl cyanide *m*-chlorophenyl hydrazone (CCCP; 0.05 μM) was titrated to uncouple ATP synthase from the electron transfer system and determine maximum electron transfer (ET) capacity. Finally, antimycin A (AMA; 2.5 μM) was added to determine residual oxygen consumption (ROX) due to oxidative side reactions (i.e., non-mitochondrial and mitochondrial non-ET pathway respiration). All respiratory states were corrected for ROX (i.e., ROX was subtracted from each state) and are presented relative to cell concentration (pmol/s/million cells).

##### T cell phenotyping

T cells subsets were identified using two flow cytometry assays. Treg, CD4^+^, and CD8^+^ cells were quantified at Baseline and 48 h. First, 1 × 10^6^ freshly isolated T cells were stained with CD45 VioBlue (Miltenyi Biotec, Cat. No. 130-110-637), CD4 VioGreen (Miltenyi Biotec, Cat. No. 130-113-230), CD25 VioBright B515 (Miltenyi Biotec, Cat. No. 130-113-287), CD127 PE (Miltenyi Biotec, Cat. No. 130-133-753), and CD8 PerCP-Vio700 (Miltenyi Biotec, Cat. No. 130-110-682) for 10 min in the dark at 4°C. Cells were washed in PBS, then fixed for 30 min with fixation buffer (Miltenyi Biotec, Cat. No. 130-122-981). Cells were centrifuged for 5 min at 300 g at 4°C, then permeabilized (Miltenyi Biotec, Cat. No. 130-122-981) along with FoxP3 VioR667 and Tandem Signal Enhancer (Miltenyi Biotec, Cat. No. 130-099-887) for 30 min at 4°C in the dark. Cells were centrifuged a final time for 5 min at 300 g, then resuspended in PBS and immediately read on a flow cytometer (CytoFlex S, Beckman Coulter). The hierarchical gating strategy is shown in Figure S2.

CD4^+^ T cell phenotypes were quantified at Baseline and 48 h. First, 1 × 10^6^ freshly isolated T cells stained with CD4 VioGreen (Miltenyi Biotec, Cat. No. 130-113-230), CD183 VioBright FITC (Miltenyi Biotec, Cat. No. 130-118-545), CD194 PE-Vio770 (Miltenyi Biotec, Cat. No. 130-118-359), CD196 PE-Vio615 (Miltenyi Biotec, Cat. No. 130-120-459), and CCR10 APC (Miltenyi Biotec, Cat. No. 130-120-406) for 10 min at 4°C in the dark. Cells were washed and resuspended in PBS for 5 min at 300 g at 4°C, incubated with 7-AAD in the dark at room temperature for 5 min, then immediately read on a flow cytometer (CytoFlex S, Beckman Coulter). The hierarchical gating strategy is shown in Figure S3.

##### T cell cytokine secretion

To quantify T cell cytokine secretion at Baseline and 48 h, a 48-well plate was incubated for 2 h at 37°C with CD3 (5 μg/ml, Invitrogen, Cat. No. 16-0037-81) to allow binding to the plate. Next, the CD3 liquid was removed, and 8 × 10^5^ freshly isolated T cells in 400 μl autologous media containing RPMI 1640, 10% autologous serum, 1× Brefeldin A (Invitrogen, Cat.No. 00-4506-51), 5 mM glucose (Sigma-Aldrich, Cat.No. G8769), and CD28 (5 μg/ml, Invitrogen, Cat.No. 16–0289) were plated and incubated for 24 h in a 5% CO_2_ incubator at 37°C.

Cells were then harvested and washed in PBS for 10 min at 300 g at 4°C. Next, cells were stained with surface stains for CD4 FITC (Miltenyi Biotec, Cat. No. 130-114-531), CCR10 PE (Miltenyi Biotec, Cat. No. 130-120-407), and a fixable viability dye (Miltenyi Biotec, Cat. No. 130-130-404) for 10 min in the dark at 4°C. Cells were washed in PBS for 5 min at 300 g at 4°C, then fixed for 30 min (Invitrogen, Cat. No. 00-8222-49). Cells were washed twice, first in PBS and then in 1X permeabilization buffer (Invitrogen, Cat. No. 00-8333-56) for 5 min at 300 g at 4°C. Next, cells were permeabilized in 1X permeabilization buffer (Invitrogen, Cat. No. 00-8333-56) for 30 min along with intracellular stains for IFNγ VioBlue (Miltenyi Biotec, Cat. No. 130-119-577) and IL-17 (Miltenyi Biotec, Cat. No. 130-118-249). Cells were washed a final time in PBS, resuspended in PBS, and immediately read on a flow cytometer (CytoFlex S, Beckman Coulter). The hierarchical gating strategy is shown in Figure S4.

#### Blood metabolites, serum cytokines and metabolic hormones

Capillary blood glucose and BHB were quantified by finger prick at every timepoint (FreeStyle Precision *Neo*, Abbott). Serum was collected at Baseline, 24 h, and 48 h. Serum was allowed to clot for 1 h, following which it was centrifuged for 15 min at 4°C and 2,000 g, aliquoted, and immediately frozen at −70°C for future batch analyses. Serum FFAs were quantified in duplicate by enzymatic assay (Wako NEFA-HR(2) Assay, FujiFilm), for which the lower and upper limits of detection are publicly available on the manufacturer’s website.

Serum insulin was quantified by absorbance in duplicate by ELISA (Cayman Chemical, Cat. No. 90095) as per the manufacturer’s directions on a plate reader (Bio-Rad). Leptin and all serum cytokines were quantified by electrochemiluminescence using the MESO QuickPlex SQ 120MM system (MesoScale Discovery): Leptin, IL-1RA, IL-6, IL-8, IL-10, FGF-21, MCP-1, and TNFα were quantified by a U-PLEX Custom Metabolic Group 1 (human) Assay (8-PLEX; MesoScale Discovery Cat.No. K151ACM) in duplicate as per the manufacturer’s instructions. GDF-15 was quantified by an R-PLEX Human GDF-15 Assay (MesoScale Discovery, Cat.No. K151YDR) in duplicate as per the manufacturer’s instructions.

#### Metabolomics

BHB-amino acids were quantified in serum using targeted metabolomics. To extract BHB-amino acids from serum for liquid chromatography-mass spectrometry (LC-MS) analysis, 150 μl of a 2:1 mixture of acetonitrile/Methanol was added to 50 μL of serum. The mixture was centrifuged at 4°C for 10 min at 15,000 rpm and the supernatant was transferred to an LC-MS vial. Targeted measurements were performed using an Agilent 6470 triple quadrupole LC-MS instrument. The MS ionization parameters for the targeted metabolomics are presented in Table S2. MS analysis was performed using AJS in negative mode. The AJS source parameters were set as follows: the dry gas temperature was set at 250°C with a gas flow of 12 L/min and the nebulizer pressure at 25nimblepsi; the sheath gas temperature was set to 300°C with the sheath gas flow set at 12 L/min; and the capillary voltage was set to 3,500 V. Separation of polar metabolites was conducted using a Luna 5 μm NH_2_ 100 LC column (Phenomenex 00G-4378-E0) with normal phase chromatography. Mobile phases were as follows: Buffer A, acetonitrile; Buffer B, 95:5 water/acetonitrile with 0.2% ammonium hydroxide and 50 mM ammonium acetate for negative ionization mode. The flow rate for each run started 100% B for 2 min at 0.7 mL/min, followed by a gradient starting at 100% B changing linearly to 50% A/50% B over the course of 18 min at 0.7 mL/min, followed by 50% A/50% B for 5 min at 0.7 mL/min. The last 5 min consisted of re-equilibration at 100% B prior to the next run. Multiple reaction monitoring was performed for the indicated metabolites with the listed dwell times, fragmentor voltage, collision energies, cell accelerator voltages and polarities (Table S2). Quantification of the endogenous metabolite concentrations were performed by generating an external standard curve with known concentrations of each metabolite. Metabolite standards were analyzed alongside the serum samples using the same targeted triple quadrupole LC/MS method in the same run. A calibration standard curve generated from the metabolite standard concentrations and total peak areas were used to calculate the concentrations of each endogenous metabolite.

#### β-hydroxybutyrylation

To isolate peripheral blood mononuclear cells (PBMCs), EDTA blood was diluted 1:1 with warm (37°C) PBS, aliquoted into a Leucosep tube (Greiner Bio-One) containing warm histopaque (Sigma-Aldrich), then centrifuged for 20 min at 800 g at 20°C. The enriched cell fraction was collected and then washed twice in warm PBS for 10 min at 300 g at 20°C. The pellet was resuspended in RBC Lysis buffer (Miltenyi Biotec) for 10 min, washed a final time in PBS, then immediately frozen at −70°C for future batch analyses.

To quantify lysine β-hydroxybutyrylation of PBMCs, isolated cells were lysed with RIPA buffer (Thermo Scientific) supplemented with protease inhibitors (Thermo Scientific). The cell lysates were sonicated using a Branson Sonifier 450 until the viscosity of lysates disappeared and then centrifuged at 14,000 rpm (17,968 ×*g*) at 4°C for 5 min to clarify cell lysates. Protein was quantified with the DC Protein Assay Kit (BioRad Laboratories). SDS-PAGE samples were prepared by mixing clarified lysates with 4× Laemmli buffer (BioRad Laboratories)/10% β-mercaptoethanol (97622, Sigma) and then heated at 95°C for 5 min. PBMCs from one randomly selected participant (FTS-14) after 48 h of fasting were used as a standard sample to ensure consistent quantification across all samples. Each sample (10 μg of protein) along with 2.5, 5, 10, and 20 μg of the standard sample were resolved by SDS-PAGE (4561086, BioRad Laboratories) and then transferred to a nitrocellulose membrane using the Trans-Blot Turbo Transfer system (BioRad Laboratories). Membranes were stained with Ponceaus S, blocked with 5% milk/TBS-T (Tris-Buffered Saline/0.1% Tween 20) for 60 min, and incubated with primary anti-Kbhb antibody (PTM-1201RM, PTM BIO), followed by secondary HRP-conjugated antibodies. Blots were developed using a chemiluminescent substrate (SuperSignal West Pico PLUS; Thermo Scientific) and imaged with an Azure 300 (Azure Biosystems). Image quantification was performed using ImageJ 1.53k. Ponceau S staining was used to normalize total protein loading across samples.

### Quantification and statistical analysis

Previous studies examining differences in T cell numbers, function, and phenotype have reported medium to large effect sies effect sizes (*d* = 0.6–0.7) when comparing lean individuals and individuals with obesity. Differences in resting whole body metabolism (e.g., metabolic flexibility, insulin resistance) between lean and obesity also typically yield medium to large effect sizes (*d* > 1). Therefore, we anticipated a medium-to-large effect size for the primary comparison between lean and obesity for T cell metabolism markers, counts, and function. Using an effect size of *d* = 1.0, a two-tailed alpha of 0.05 and 80% power, *N* = 17 per group was estimated for an independent sample *t*-test (G∗Power v3.1). This sample size provided 86% power to detect a significant interaction (alpha 0.05) for a medium effect size (*f* = 0.25) for the 2 group × 2 timepoint design in the 48-fasting intervention, assuming a repeated measures correlation of r = 0.8. A medium effect size was anticipated based on the medium-to-large effect size seen in studies showing that fasting impacts T cell numbers (*d* = 0.8–1.0), using means and standard deviations and a repeated measures correlation of r = 0.8. To obtain balanced numbers of males and females in each group, we recruited *N* = 16 per group with 8 males and 8 females per group.

All statistical analyses were performed in R Studio (Version 4.0.3) using the sjPlot, lme4, lmerTest, and emmeans packages.^67^^,^^68^^,^^69^^,^^70^^,^^71^ Prior to analysis, data were visually inspected for normality and linearity using a Q-Q plot, and homoscedasticity was assessed by plotting residuals against fitted values. Cook’s distance was used to assess the presence of influential data points. A Cook’s distance above the threshold, calculated as 4/N, was considered influential, where N represents the number of participants included in the dataset. These values were then checked but only excluded if there was sufficient reason to do so (i.e., technical error, physiological implausibility, or if inclusion significantly changed the outcome or interpretation of the statistical test). All outcomes were analyzed using a linear mixed model (LMM). The LMM was built with a fixed effect for time and group, and a random slope for participant. Significance was set at *p* < 0.05. All data are reported as estimated marginal means with 95% confidence intervals as derived from the LMM unless indicated otherwise. Significant within-factor comparisons with Bonferroni correction for multiple comparisons are only reported if the main effect or interaction was significant. All estimated marginal means with 95% confidence intervals are reported in Tables S3–S7 and data are reported disaggregated by sex as means with standard deviations in Tables S8–S12. Details of each statistical test are reported in the figure legends.

### Additional resources

Any estimated marginal mean with 95% confidence interval not reported in the paper, as well as all data disaggregated by sex, is located in the Supplemental Materials.

This trial was registered on ClinicalTrials.gov (NCT05886738).
