# Supplementary material for: Altered immunometabolic response to fasting in humans living with obesity
Source: iScience. 2025 Jun 11;28(7):112872. doi: 10.1016/j.isci.2025.112872 (PMC12256293; doi:10.1016/j.isci.2025.112872)
Supplement: Document S1. Figures S1–S4, Tables S1–S12, and Data S1 [file mmc1.pdf]

iScience, Volume *28*

Supplemental information

Altered immunometabolic response to fasting in humans living with obesity

Helena Neudorf, Roderick E. Sandilands, Spencer Ursel, Hillary Shaba, Darren Barg, Takeshi Tsusaka, María Dolores Moya-Garzón, Erica Vaz, Patricia Schimweg, Emily L. Goldberg, Jonathan Z. Long, Karsten Krüger, Hashim Islam, and Jonathan P. Little

Supplemental Figures

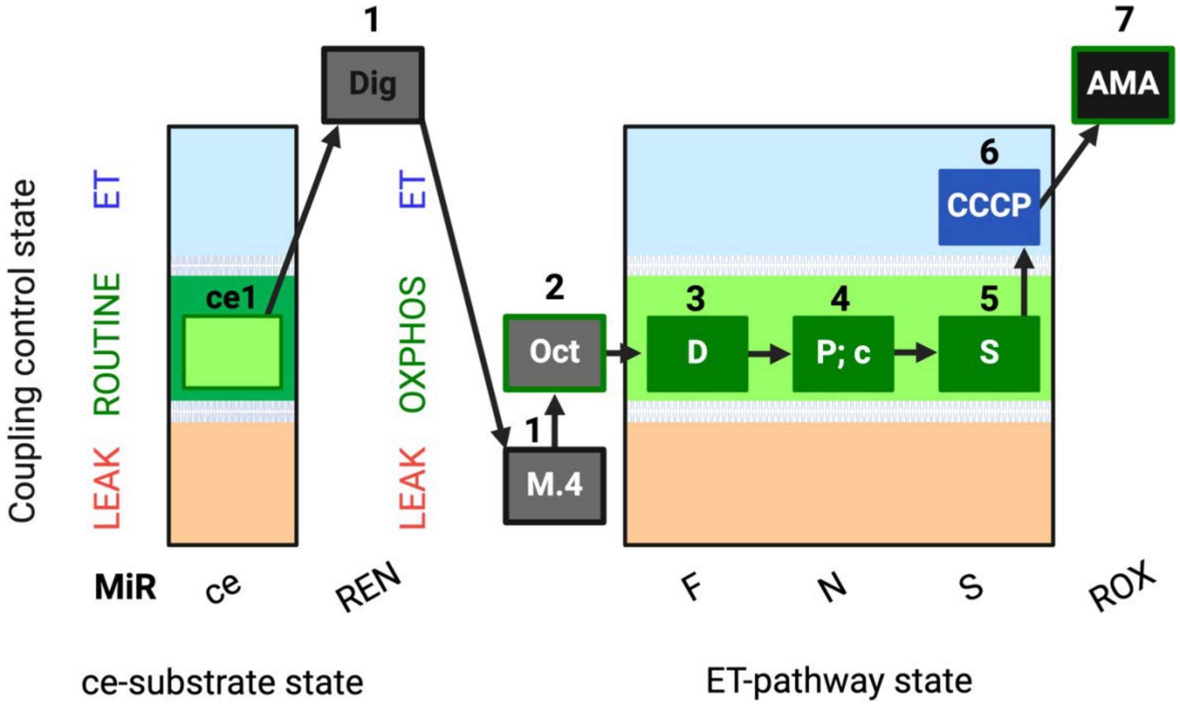

Figure S1. Substrate-uncoupler-inhibitor titration (SUIT) protocol for characterization of T cell mitochondrial metabolism. Cells (ce) were suspended in mitochondrial respiration medium (MiR) and permeabilized with digitonin (Dig). Next malate (M), octanoylcarnitine (Oct) were added. ADP (D) was added to stimulate fat-supported oxidative phosphorylation, followed by pyruvate (P) to stimulate carbohydrate-supported respiration through complex I, and cytochrome C (c) to determine mitochondrial outer membrane integrity. Succinate (S) was added to achieve maximal oxidative phosphorylation capacity through CI and CII. Carbonyl cyanide m- chlorophenyl hydrazone (CCCP) was titrated to uncouple ATP synthase from the electron transfer (ET) system and determine the maximum ET capacity. Finally, antimycin A (AMA) was added to determine residual oxygen consumption (ROX) due to oxidative side reactions. REN, residual endogenous oxygen consumption.

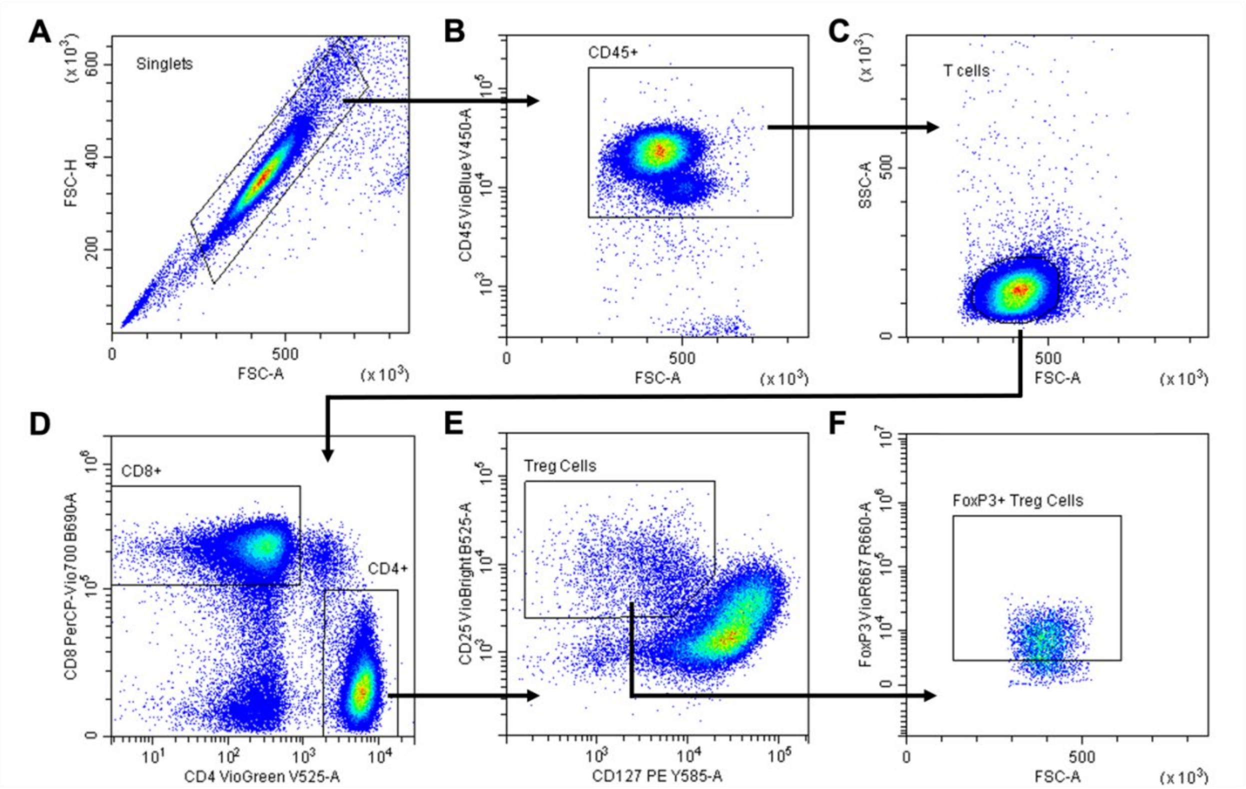

Figure S2. Hierarchical gating strategy for determination of CD4+ cells, CD8+ T cells, T regulatory (Treg) cells, and FoxP3 expression in Treg cells from human T cells isolated from whole blood. A) Singlets were identified, then **B)** $CD45^+$ T cells were **C)** selected based on characteristic scatter profile. **D)** $CD4^+$ and $CD8^+$ T cell subsets were identified by positive or negative staining for CD4 and CD8, respectively. **E)** $CD4^+$ T cells which exhibited bright staining for CD25 and low or negative staining for CD127 were identified as Treg cells, and **E)** FoxP3 expression was quantified in these $CD4^+/CD25^{hi}/CD127^{dim/-}$ cells.

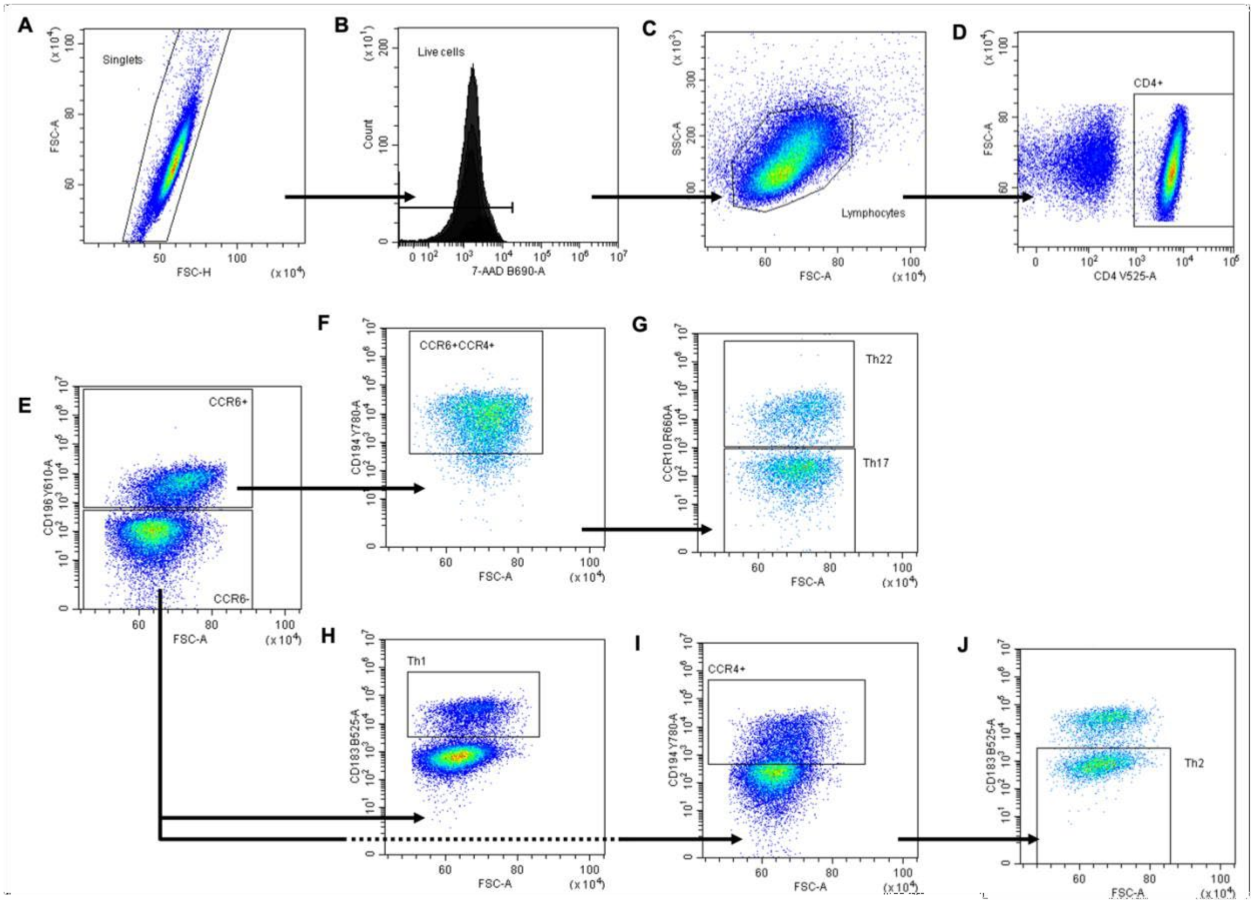

Figure S3. Hierarchical gating strategy for determination of CD4⁺ T cell phenotype from human T cells isolated from whole blood. A) Singlets and then **B)** live cells (7-AAD⁺) were selected. **C)** Lymphocytes were selected based on characteristics scatter profile and **D)** CD4⁺ T cells were selected. CD4⁺ T cell subsets were identified as follows: Th1 cells were identified based on **E)** CCR6 (CD196) negative staining and **H)** CD183 positive staining. Th2 cells were identified based on **E)** CCR6 (CD196) negative staining, **I)** CCR4 (CD194) positive staining, and **J)** CD183 negative staining. Th17 cells were identified by **E)** CCR6 (CD196) positive staining, **F)** CCR4 (CD194) positive staining and **G)** negative CCR10 staining. Th22 cells were identified based on **E)** CCR6 (CD196) positive staining, **F)** CCR4 (194) positive staining and **G)** positive CCR10 staining.

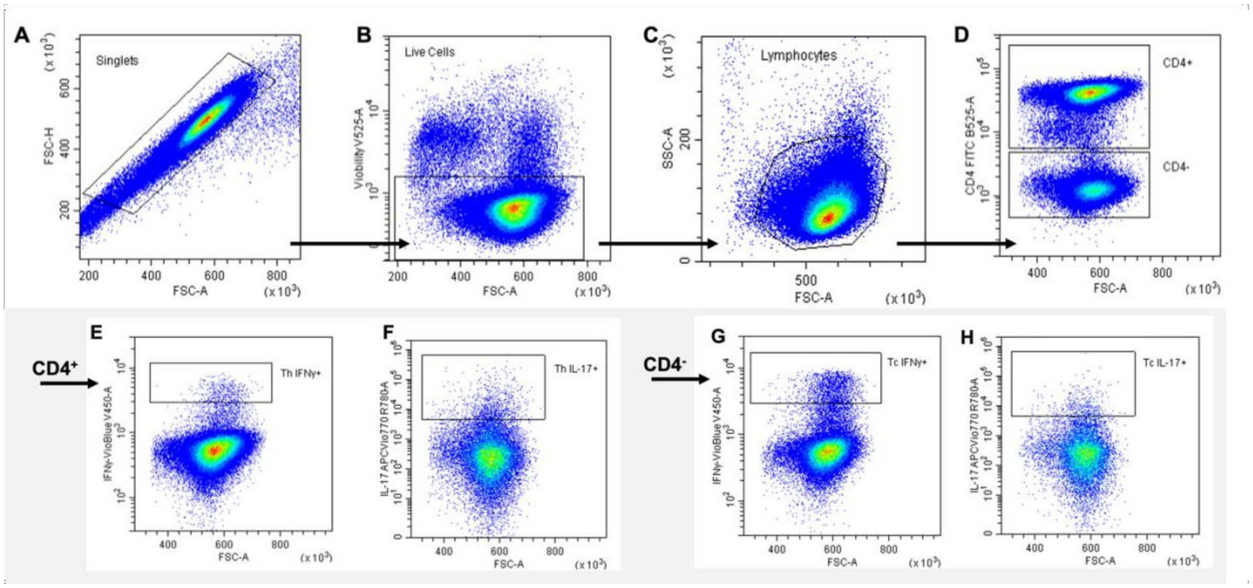

Figure S4. **Hierarchical gating strategy for determination of intracellular cytokine secretion in isolated CD4⁺ and CD4⁻ human T cells following a 24-hour activation culture. A)** Singlets were identified, then **B)** live cells with **C)** scatter profile characteristic of T cells were selected. **D)** CD4⁺ and CD4⁻ (presumed to be CD8⁺) T cells were selected and secretion of IFNγ **(E & G)**, and IL-17 **(F & H)** were quantified.

Supplemental Tables

Table S1. Macronutrient and micronutrient composition of the Ensure Plus Calories breakfast drink (Abbott).

	Amount Per Bottle (235 mL)
Calories (kcal)	350
Total Fat (g)	11
Saturated Fat (g)	1
Trans Fat (g)	0
Polyunsaturated Fat (g)	4
Monounsaturated Fat (g)	5
Cholesterol (mg)	10
Sodium (mg)	210
Carbohydrate (g)	50
Fibre (g)	0
Sugars (g)	20
Protein (g)	13
Cholesterol (mg)	10
Sodium (mg)	210

Table S2. LC-MS parameters for metabolites measured.

Metabolite	Precursor Ion	MS1 Res	Product Ion	MS2 Res	Dwell	Fragmentor	Collision Energy	Cell Accelerator Voltage	Retention time (min)	Polarity
BHB-Phe	250.1	Unit	164.1	Unit	50	135	10	5	12.5	Negative
BHB-Val	202.1	Unit	116.1	Unit	50	135	10	5	13	Negative
BHB-(Iso)Leu	216.1	Unit	130.1	Unit	50	135	10	5	12.6	Negative
BHB-Met	234.1	Unit	148.0	Unit	50	135	10	5	12.9	Negative

Table S3. Baseline participant characteristics.

	L-BMI			O-BMI			P-value
	All (N = 16)	Males (N = 8)	Females (N = 8)	All (N = 16)	Males (N = 8)	Females (N = 8)	
Age (years)	27 (11)	27 (9)	28 (13)	38 (13)	32 (11)	43 (13)	*0.020
BMI (kg/m^2)	22.4 (2.7)	23.6 (2.8)	21.1 (1.8)	35 (5.2)	35.5 (5.0)	34.8 (5.8)	*<0.001
Body fat (%)	20.5 (6.5)	17.5 (5.8)	23.9 (5.7)	38.9 (8.0)	33.6 (5.7)	44.1 (6.3)	*<0.001
Fat mass (kg)	13.7 (5.5)	13.6 (5.9)	14.0 (5.4)	41.0 (13.5)	39.3 (14.3)	42.7 (13.4)	*<0.001
Fat-free mass (kg)	49.8 (13.4)	55.6 (16.5)	43.1 (1.9)	63.6 (13.3)	75.3 (6.6)	52.0 (5.0)	*0.017
SBP (mmHg)	110 (13)	114 (13)	104 (13)	127 (14)	131 (13)	123 (15)	*0.003
DBP (mmHg)	72 (9)	72 (8)	72 (11)	83 (9)	83 (9)	84 (10)	*0.001
Pulse (mmHg)	66 (11)	64 (10)	69 (12)	68 (13)	69 (16)	68 (13)	0.608

Data are means (SD). BMI, body mass index; DBP, diastolic blood pressure; SBP, systolic blood pressure. * $P < 0.05$ from a paired T-test comparing L-BMI to O-BMI.

Table S4. Systemic metabolites and hormones through a 48-hour fast.

	L-BMI			O-BMI			P-value		
	Baseline	24 hours	48 hours	Baseline	24 hours	48 hours	Main Effect of Time	Main Effect of Group	Group x Time Interaction
FFA (mM)	0.16 [0.03, 0.30]	0.98 [0.85, 1.12]	1.16 [1.02, 1.29]	0.20 [0.07, 0.33]	0.85 [0.71, 0.99]	1.03 [0.90, 1.17]	*<0.001	0.269	0.277
BHB (mM)	0.1 [-0.2, 0.5]	1.4 [1.1, 1.8]	3.4 [3.1, 3.7]	0.1 [0.0, 0.4]	0.5 [0.2, 0.9]	1.6 [1.2, 1.9]	*<0.001	*<0.001	*<0.001
BHB-leucine (nM)	2.2 [-2.0,6.5]	-	25.5 [21.3,29.8]	2.0 [-2.0,6.3]	-	8.9 [4.5,13.3]	*<0.001	*<0.001	*<0.001
BHB-valine (nM)	2.0 [-2.5,6.5]	-	34.0 [29.5,38.5]	2.8 [-1.7,7.3]	-	11.2 [6.6,15.9]	*<0.001	*<0.001	*<0.001
BHB-methionine (nM)	2.1 [1.4,2.9]	-	6.5 [5.2,6.7]	2.0 [1.3,2.8]	-	3.3 [2.5,4.1]	*<0.001	*<0.004	*<0.001
BHB-phenylalanine (nM)	16.5 [-0.7,33.7]	-	31.7 [15.0,48.4]	43.5 [26.3,60.7]	-	32.4 [14.6,50.2]	0.795	0.102	0.129
Glucose (mM)	5.1 [4.8, 5.4]	4.0 [3.7, 4.3]	3.4 [3.1, 3.7]	5.1 [4.8, 4.7]	4.4 [4.1, 4.7]	4.0 [3.7, 4.3]	*<0.001	*0.022	*0.029
Insulin (pmol/L)	69.1 [43.1, 95.1]	9.2 [-16.9 35.1]	9.9 [0.0, 36.6]	134 [108.0, 160.0]	34.8 [7.3, 62.3]	25.4 [-1.3, 52.1]	*<0.001	*0.015	0.065
Leptin (ng/mL)	803 [-1,355, 2,961]	-	54.6 [-2,104, 2,213]	9,519 [7,361, 11,677]	-	3,173 [974, 5,371]	*<0.001	*<0.001	*<0.001

BHB, beta-hydroxybutyrate; FFA, free fatty acids. Data are estimated marginal means [95 % confidence interval] derived from a linear mixed effects model. *P < 0.05.

Table S5. Mitochondrial respiratory states measured in isolated human T cells.

	L-BMI		O-BMI		P-value		
	Baseline	48 hours	Baseline	48 hours	Main effect of Time	Main effect of Group	Group x Time Interaction
Routine respiration (pmol/[s x 10^6 cells])	3.8 [3.3,4.2]	3.2 [2.8,3.6]	3.9 [3.4,4.3]	3.1 [2.6,3.5]	*<0.001	0.958	0.496
Leak respiration (pmol/[s x 10^6 cells])	3.2 [2.9,3.6]	2.8 [2.4,3.2]	3.1 [2.7,3.5]	2.4 [2.0,2.8]	*0.004	0.16	0.450
Fat-supported respiration (pmol/[s x 10^6 cells])	5.2 [4.6,5.8]	4.4 [3.8,5.0]	6.4 [5.4,6.8]	4.8 [4.2,5.5]	*<0.001	0.78	0.387
Fat+CI-supported respiration (pmol/[sx10^6 cells])	5.7 [4.9,6.4]	4.5 [3.8,5.2]	7.0 [6.2,7.8]	5.8 [5.1,6.5]	*0.003	*0.003	0.964
Maximum oxidative capacity (pmol/[s x 10^6 cells])	7.4 [6.5,8.4]	6.1 [5.1,7.1]	10.3 [9.3,11.4]	9.3 [8.4,10.3]	*0.024	*<0.001	0.735
Uncoupled respiration (pmol/[s x 10^6 cells])	12.3 [10.9,13.7]	9.5 [8.1,10.9]	14.6 [13.1,16.1]	12.2 [10.8,13.6]	*<0.001	*0.008	0.698
Residual oxygen consumption (pmol/[s x 10^6 cells])	0.6 [0.2,1.1]	1.32 [0.9,1.8]	1.0 [0.5,1.4]	1.4 [0.9,1.8]	*0.004	0.426	0.372
Fat contribution (% of maximum oxidative capacity)	70.7 [66.3,75.1]	74.5 [70.1,78.9]	59.5 [54.8,64.2]	55.0 [50.4,59.5]	0.838	*<0.001	*0.032

Data are estimated marginal means [95 % confidence interval] derived from a linear mixed effects model. *P < 0.05.

Table S6. Cell counts and T cell subsets.

	L-BMI			O-BMI			P-value		
	Baseline	24 hours	48 hours	Baseline	24 hours	48 hours	Main Effect of Time	Main Effect of Group	Group x Time Interaction
WBC (x10^3/uL)	5.7 [4.8,6.5]	6.6 [5.7,7.4]	5.6 [4.8,6.4]	6.9 [6.1,7.7]	6.3 [5.5,7.2]	7.2 [6.4,8.0]	0.730	0.095	*0.002
Lymphocyte Count (x10^3/uL)	1.7 [1.5,1.9]	1.5 [1.3,1.7]	1.4 [1.2,1.6]	1.9 [1.7,2.1]	1.8 [1.6,2.0]	1.5 [1.3,1.7]	*<0.001	0.088	0.768
Lymphocytes (% of WBC)	30.2 [26.2,34.3]	25.2 [21.1,29.2]	25.4 [21.5,29.4]	29.1 [25.2,33.1]	29.6 [25.5,33.7]	23.3 [19.3,27.2]	*<0.001	0.880	*0.013
$CD4^+$ T cells (% T cells)	58.4 [53.4,63.5]	-	58.5 [53.5,63.6]	61.1 [56.0,66.2]	-	61.5 [56.5,66.5]	0.817	0.405	0.884
$CD8^+$ T cells (% T cells)	27.0 [23.0,31.0]	-	25.2 [21.2,29.2]	26.4 [22.3,30.4]	-	26.0 [22.0,30.0]	0.389	0.962	0.563
$CD4^-$/$CD8^-$ T cells (% of T cells)	10.5 [8.1,12.9]	-	12.8 [10.4,15.2]	10.0 [7.6,12.5]	-	9.3 [6.9,11.7]	0.316	0.190	0.059
Th1 Cells (% of $CD4^+$ T cells)	16.7 [13.4,20.1]	-	15.3 [12.0,18.7]	19.8 [16.5,23.1]	-	20.8 [17.5,24.1]	0.801	0.061	0.153
Th2 Cells (% of $CD4^+$ T cells)	9.9 [8.5,11.3]	-	10.0 [8.6,11.4]	9.2 [7.8,10.6]	-	10.0 [8.5,11.4]	0.341	0.689	0.456
Th17 Cells (% of $CD4^+$ T cells)	11.0 [9.3,12.7]	-	10.6 [8.8,12.4]	13.5 [11.7,15.3]	-	13.7 [12.0,15.5]	0.930	*0.023	0.441
Th22 Cells (% of $CD4^+$ T cells)	4.3 [3.5,5.0]	-	4.2[3.5,5.0]	5.0 [4.2,5.8]	-	4.8 [4.0,5.6]	0.539	0.235	0.631
Treg Cells	8.9 [7.7,10.1]	-	8.5 [7.3,9.7]	7.5 [6.4,8.7]	-	7.3 [6.1,8.5]	0.340	0.101	0.812

Data are estimated marginal means [95 % confidence interval] derived from a linear mixed effects model. WBC, white blood cells. *P < 0.05.

Table S7. Markers of T cell function.

	L-BMI		O-BMI		P-value		
	Baseline	48 hours	Baseline	48 hours	Main effect of Time	Main effect of Group	Group x Time Interaction
CD4 Expression (MFI, a.u.)	5433 [5172,5694]	4979 [4718,5240]	5727 [5463,5992]	5691 [5430,5952]	*<0.001	*0.006	*0.004
CD8 Expression (MFI, a.u.)	235120 [215681,254559]	200764 [181325,220204]	246473 [226396,266550]	226790 [207351,246229]	*0.008	0.061	0.457
$CD4^+$ T cells Intracellular IFNγ (MFI, a.u.)	4049 [3863,4235]	4131 [3916,4345]	3813 [3598,4027]	3878 [3654,4103]	0.462	*0.035	0.936
$CD8^+$ T cells Intracellular IFNγ (MFI, a.u.)	4668 [4467,4870]	4747 [4516,4978]	4469 [4236,4702]	4458 [4215,4700]	0.743	0.060	0.660
$CD4^+$ T cells Intracellular IL-17 (MFI, a.u.)	20599 [12856,28342]	20330 [11766,28893]	25389 [15620,35158]	19512 [9227,29797]	0.473	0.684	0.512
$CD8^+$ T cells Intracellular IL-17 (MFI, a.u.)	11174 [600,21749]	16516 [5522,27510]	23856 [12839,34872]	36239 [24202,48276]	0.102	*0.013	0.503
Δ Normalized Kbhb (a.u. x 10^{-3})	0.3 [-8.9,9.4]	42.3 [33.2,51.4]	0.1 [-8.318.5]	23.3 [14.9,31.7]	*<0.001	*0.049	*0.031

Data are estimated marginal means [95 % confidence interval] derived from a linear mixed effects model. *P < 0.05.

Table S8. Body characteristic changes throughout a 48-hour fast.

	Lean (N = 16)			Obesity (N = 16)		
	**Baseline**	**24 hours**	**48 hours**	**Baseline**	**24 hours**	**48 hours**
**Males (N = 8 per group)**						
Weight (kg)	74.7 (12.2)	73.9 (12.2)	71.7 (12.7)	114.6 (19.2)	112.4 (19.0)	110.9 (18.7)
BMI (kg/m^2)	23.6 (2.8)	23.4 (2.9)	23.0 (3.1)	35.5 (5.0)	34.7 (5.0)	34.3 (4.9)
Body fat (%)	17.5 (5.8)	17.5 (6.5)	17.4 (6.8)	33.6 (5.7)	35.6 (7.0)	36.7 (8.4)
Fat mass (kg)	13.6 (5.9)	13.5 (6.4)	13.1 (6.6)	39.3 (14.3)	41.1 (16.4)	41.9 (18.4)
Fat-free mass (kg)	61.2 (7.3)	60.4 (7.4)	58.6 (6.9)	75.3 (6.6)	71.3 (6.0)	69.0 (5.5)
SBP (mmHg)	114 (13)	119 (7)	118 (11)	131 (13)	129 (10)	129 (14)
DBP (mmHg)	72 (8)	71 (7)	69 (8)	83 (9)	86 (10)	81 (6)
Pulse (bpm)	64 (10)	68 (13)	74 (10)	69 (16)	72 (16)	78 (14)
**Females (N = 8 per group)**						
Weight (kg)	56.9 (6.5)	56.6 (7.4)	55.0 (6.9)	94.7 (17.5)	93.6 (16.9)	92.4 (16.9)
BMI (kg/m^2)	20.8 (1.9)	20.5 (2.1)	20.1 (2.0)	34.8 (5.8)	34.4 (5.6)	34.0 (5.5)
Body fat (%)	24.2 (5.3)	25.1 (5.3)	24.5 (5.0)	44.1 (6.3)	44.4 (6.3)	44.6 (6.7)
Fat mass (kg)	14.0 (5.0)	14.5 (5.2)	13.8 (4.8)	42.7 (13.4)	42.4 (13.2)	42.2 (13.4)
Fat-free mass (kg)	42.9 (1.8)	42.1 (2.2)	41.3 (2.3)	52.0 (5.0)	51.2 (4.7)	50.3 (4.4)
SBP (mmHg)	108 (15)	101 (10)	107 (14)	123 (15)	120 (15)	117 (11)
DBP (mmHg)	73 (10)	68 (10)	73 (11)	84 (10)	83 (11)	80 (13)
Pulse (bpm)	69 (11)	72 (14)	81 (14)	68 (13)	65 (12)	68 (15)

Data are means (SD). BMI, body mass index; DBP, diastolic blood pressure; SBP, systolic blood pressure.

Table S9. Measures of systemic metabolism throughout a 48-hour fast.

	L-BMI (N = 16)			O-BMI (N = 16)		
	**Baseline**	**24 hours**	**48 hours**	**Baseline**	**24 hours**	**48 hours**
**Males (N = 8 per group)**						
RER (a.u.)	0.87 (0.07)	0.79 (0.03)	0.76 (0.03)	0.85 (0.08)	0.79 (0.04)	0.75 (0.05)
REE (kcal/min)	1.33 (0.25)	1.40 (0.23)	1.43 (0.25)	1.62 (0.23)	1.55 (0.19)	1.64 (0.14)
Carbohydrate oxidation (g/min)	0.18 (0.08)	0.09 (0.03)	0.07 (0.04)	0.20 (0.11)	0.11 (0.06)	0.06 (0.07)
Fat oxidation (g/min)	0.06 (0.03)	0.11 (0.03)	0.12 (0.03)	0.08 (0.04)	0.11 (0.01)	0.15 (0.03)
FFA (mM)	0.17 (0.08)	0.93 (0.32)	1.05 (0.33)	0.22 (0.09)	0.77 (0.20)	0.96 (0.36)
BHB (mM)	0.1 (0.1)	1.7 (1.1)	3.5 (1.2)	0.09 (0.04)	0.5 (0.2)	1.6 (0.8)
Glucose (mM)	5.0 (0.8)	3.8 (0.4	3.4 (0.2)	5.2 (0.8)	4.5 (0.4)	4.2 (0.4)
Insulin (pmol/L)	65.8 (38.4)	8.5 (3.2)	12.0 (4.1)	152.7 (105.8)	41.6 (28.2)	27.4 (23.3)
Leptin (ng/mL)	650 (868)	-	64 (83)	6,215 (3,936)	-	2,125 (3,498)
**Females (N = 8 per group)**						
RER (a.u.)	0.87 (0.03)	0.80 (0.06)	0.75 (0.03)	0.84 (0.07)	0.79 (0.05)	0.75 (0.05)
REE (kcal/min)	1.11 (0.17)	0.87 (0.35)	0.92 (0.25)	1.62 (0.22)	1.55 (0.17)	1.65 (0.15)
Carbohydrate oxidation (g/min)	0.15 (0.03)	0.05 (0.02)	0.04 (0.03)	0.19 (0.10)	0.11 (0.07)	0.05 (0.06)
Fat oxidation (g/min)	0.05 (0.02)	0.07 (0.03)	0.08 (0.02)	0.09 (0.04)	0.12 (0.03)	0.15 (0.03)
FFA (mM)	0.16 (0.07)	1.04 (0.40)	1.29 (0.39)	0.18 (0.04)	0.88 (0.17)	1.07 (0.28)
BHB (mM)	0.2 (0.1)	1.1 (0.6)	3.3 (1.2)	0.09 (0.04)	0.6 (0.3)	1.4 (0.7)

Glucose (mM)	5.0 (0.2)	4.2 (0.4)	3.3 (0.6)	4.9 (0.9)	4.3 (0.4)	3.8 (0.5)
Insulin (pmol/L)	71.7 (36.7)	9.9 (4.6)	7.4 (3.3)	129.2 (128.2)	34.8 (43.4)	22.1 (20.0)
Leptin (ng/mL)	1,195 (1,724)	-	45 (55)	13,767 (8,946)	-	4,525 (4,571)

Data are means (SD). BHB, beta-hydroxybutyrate; FFA, free fatty acids; REE, resting energy expenditure; RER, respiratory exchange ratio.

Table S10. Measures of T cell metabolism before and after a 48-hour fast.

	Lean (N = 16)		Obesity (N = 16)	
	**Baseline**	**48hrs**	**Baseline**	**48hrs**
**Males (N = 8 per group)**				
Routine respiration (pmol/[s x 10^6 cells])	3.5 (0.9)	2.9 (0.7)	3.9 (0.2)	3.2 (0.4)
Leak respiration (pmol/[s x 10^6 cells])	3.0 (1.0)	2.7 (0.4)	2.9 (0.4)	2.3 (0.6)
Fat-supported respiration (pmol/[s x 10^6 cells])	5.2 (1.5)	4.4 (1.1)	6.1 (0.7)	4.5 (1.4)
Fat+CI-supported respiration (pmol/[sx10^6 cells])	5.7 (1.8)	4.6 (1.1)	6.3 (0.5)	5.2 (1.3)
Fat+CI+CII (pmol/[s x 10^6 cells])	7.6 (1.9)	6.0 (1.2)	9.9 (1.0)	8.6 (2.1)
Uncoupled Respiration (pmol/[s x 10^6 cells])	11.4 (3.4)	8.5 (2.0)	12.3 (1.2)	11.3 (1.7)
Residual Oxygen Consumption (pmol/[s x 10^6 cells])	0.8 (0.5)	1.2 (0.7)	0.9 (0.9)	1.4 (0.8)
**Females (N = 8 per group)**				
Routine respiration (pmol/[s x 10^6 cells])	4.0 (1.0)	3.5 (0.6)	3.9 (1.1)	2.9 (1.2)
Leak respiration (pmol/[s x 10^6 cells])	3.5 (0.9)	2.9 (0.6)	3.3 (1.0)	2.5 (1.0)
Fat-supported respiration (pmol/[s x 10^6 cells])	5.2 (1.2)	4.4 (0.6)	6.3 (1.9)	5.1 (1.5)
Fat+CI-supported respiration (pmol/[sx10^6 cells])	5.6 (1.5)	4.5 (0.7)	7.6 (2.2)	6.4 (1.3)
Fat+CI+CII (pmol/[s x 10^6 cells])	7.3 (1.8)	6.2 (1.6)	10.7 (2.8)	10.0 (2.3)

Uncoupled Respiration (pmol/[s x 10^6 cells])	13.2 (3.4)	10.6 (2.6)	16.3 (3.6)	13.1 (1.5)
Residual Oxygen Consumption (pmol/[s x 10^6 cells])	0.4 (0.9)	1.3 (1.2)	1.0 (1.0)	1.8 (1.4)

Data are means (SD).

Table S11. Cell counts and proportions throughout a 48-hour fast.

	Lean (N = 16)			Obesity (N = 16)		
	**Baseline**	**24hrs**	**48hrs**	**Baseline**	**24hrs**	**48hrs**
**Males (N = 8 per group)**						
WBC (x10^3/ul)	6.16 (1.66)	7.18 (2.09)	5.87 (1.82)	6.66 (1.77)	6.34 (2.23)	7.35 (2.31)
Lymphocytes (%)	27.9 (7.3)	21.3 (9.8)	25.0 (7.5)	31.7 (9.1)	30.8 (7.8)	26.0 (8.0)
Lymphocytes (x10^3/ul)	1.65 (0.29)	1.41 (0.50)	1.41 (0.48)	1.99 (0.36)	1.83 (0.34)	1.85 (0.73)
CD4$^+$ T cells (% of total T cells)	*64.2 (11.4)	--	*68.3 (10.8)	60.0 (6.1)	--	60.7 (8.4)
CD8$^+$ T cells (% of total T cells)	*28.8 (5.4)	--	*29.0 (8.7)	29.0 (5.9)	--	26.9 (5.7)
Th1 (% of CD4$^+$ T cells)	*14.9 (4.1)	--	*15.2 (4.7)	16.6 (5.6)	--	18.2 (4.2)
Th2 (% of CD4$^+$ T cells)	*15.0 (12.0)	--	*11.4 (2.4)	11.8 (7.9)	--	13.2 (7.9)
Th17 (% of CD4$^+$ T cells)	*10.6 (2.0)	--	*9.3 (2.5)	15.8 (7.2)	--	13.6 (4.0)
Th22 (% of CD4$^+$ T cells)	*4.5 (1.2)	--	*4.4 (1.2)	5.6 (1.2)	--	5.5 (1.0)
Treg (% of CD4$^+$ T cells)	*11.2 (1.9)	--	*12.7 (4.6)	8.8 (2.2)	--	7.4 (1.9)
**Females (N = 8 per group)**						
WBC (x10^3/ul)	5.68 (1.52)	6.15 (2.14)	5.56 (1.16)	7.59 (0.51)	7.11 (0.13)	7.37 (0.89)
Lymphocytes (%)	30.8 (6.3)	27.8 (10.5)	25.9 (9.0)	27.1 (8.2)	25.7 (3.4)	21.1 (4.9)
Lymphocytes (x10^3/ul)	1.70 (0.40)	1.56 (0.59)	1.37 (0.34)	2.08 (0.76)	1.83 (0.27)	1.54 (0.18)

$CD4^+$ T cells (% of total T cells)	†65.0 (13.3)	--	†69.2 (11.6)	61.7 (8.4)	--	62.3 (7.8)
$CD8^+$ T cells (% of total T cells)	†28.1 (13.2)	--	†26.7 (12.6)	27.5 (8.5)	--	25.2 (7.6)
Th1 (% of $CD4^+$ T cells)	†15.3 (3.1)	--	†16.6 (4.1)	22.6 (8.8)	--	23.0 (8.9)
Th2 (% of $CD4^+$ T cells)	†9.1 (1.5)	--	†13.3 (6.6)	9.3 (3.6)	--	11.8 (6.5)
Th17 (% of $CD4^+$ T cells)	†12.6 (4.2)	--	†12.9 (3.0)	13.6 (4.2)	--	13.9 (3.7)
Th22 (% of $CD4^+$ T cells)	†4.8 (2.0)	--	†4.6 (1.4)	5.3 (3.0)	--	5.1 (3.1)
Treg (% of $CD4^+$ T cells)	9.4 (2.6)	--	9.6 (1.9)	6.4 (2.3)	--	62.3 (3.3)

Data are means (SD). CD, cluster of differentiation; HCT, hematocrit; HGB, hemoglobin; RBC, red bloods cells; Th, T helper cell; Treg, T regulatory cell; WBC, white blood cells. *N = 7, †N = 6.

Table S12. Measures of T cell function before and after a 48-hour fast.

	Lean (N = 16)		Obesity (N = 16)	
	**Baseline**	**48hrs**	**Baseline**	**48hrs**
**Males (N = 8 per group)**				
CD4 receptor MFI (a.u.)	*5,361 (371)	*4,949 (417)	5,617 (599)	5,670 (620)
CD8 receptor MFI (a.u.)	*234,290 (30,070)	*196,882 (23,576)	224,457 (53,097)	228,180 (31,773)
$CD4^+$ T cell IFNγ MFI (a.u.)	4,115 (226)	4,197 (491)	*3,869 (418)	*3,799 (361)
$CD8^+$ T cell IFNγ MFI (a.u.)	4,696 (554)	4,803 (207)	*4,511 (480)	*4,337 (486)
$CD4^+$ T cell IL-17 MFI (a.u.)	14,827 (7,319)	15,587 (5,980)	*26,569 (17,780)	*58,296 (98,999)
$CD8^+$ T cell IL-17 MFI (a.u.)	11,249 (7,413)	16,249 (12,548)	*23,732 (25,077)	*23,687 (20,122)
**Females (N = 8 per group)**				
CD4 receptor MFI (a.u.)	5,491 (413)	5,168 (545)	5,792 (552)	5,712 (505)
CD8 receptor MFI (a.u.)	229,836 (31,907)	196,141 (9,970)	265,736 (44,490)	225,400 (44,304)
$CD4^+$ T cell IFNγ MFI (a.u.)	3,983 (94)	4,091 (238)	†3,745 (640)	†3,989 (470)
$CD8^+$ T cell IFNγ MFI (a.u.)	4,641 (131)	4,725 (211)	†4,411 9579)	†4,578 (414)
$CD4^+$ T cell IL-17 MFI (a.u.)	26,372 (19,662)	23,728 (20,353)	†75,462 (115,655)	†34,207 (30,038)
$CD4^+$ T cell IL-17 MFI (a.u.)	10,740 (4,278)	32,480 (47,866)	†22,572 (14,011)	†47,895 (32,659)

Data are means (SD). A.u., arbitrary units; CD, cluster of differentiation; IFN, interferon; IL, interleukin; MFI, median fluorescence intensity. *N = 7. †N = 6.

Title: Investigating the effect of short-term fasting on T cell metabolism, function, and phenotype in obesity

Research question: Does a 48-hour fast differentially impact the metabolism, phenotype, and function of T cells derived from lean individuals versus people with obesity?

Aims

1) To determine if T cell mitochondrial bioenergetics differs between lean individuals and individuals with obesity
2) To examine how 48 hours of fasting impacts T cell numbers, T cell functions, and T cell mitochondrial bioenergetics in lean individuals and individuals living with obesity
3) To compare $CD4^+$ and $CD8^+$ T cell metabolism and mitochondrial bioenergetics during 48 hours of fasting between people with obesity versus lean individuals.
4) To compare how 48 hours of fasting impacts circulating T $CD4^+$ and $CD8^+$ cell subsets/phenotype between people with obesity versus lean individuals.
5) To compare how 48 hours of fasting impacts circulating $CD4^+$ and $CD8^+$ T cell suppressor and effector cell functioning.

Hypotheses

1) T cells from lean participants will have higher maximal mitochondrial respiration and increased ability to oxidize fatty acids when compared to T cells from individuals with obesity
2) Fasting for 48 hours will increase T regulatory cells, $T_{H/C}$ 2, $T_{H/C}$ 22 and decrease in $T_{H/C}$ 1, $T_{H/C}$ 17 effector T cells
3) Lean participants will exhibit a greater increase in $CD4^+$ and $CD8^+$ T cell mitochondrial oxidative capacity and fat oxidation vs. individuals with obesity.
4) Lean participants will exhibit a greater increase in T regulatory cells, Th/c2, Th/c22 and decrease in Th/c1, Th/c17 effector T cells vs. individuals with obesity.
5) Lean participants will exhibit a greater increase in T regulatory cell suppressive function vs. individuals with obesity.

BACKGROUND & RATIONALE

Obesity affects nearly one third of Canadians (1) and this excess adiposity is associated with negative impacts on health including dysregulation of the immune system (2, 3). This results in a weakened defense against pathogens such that individuals with obesity exhibit weaker responses to vaccination and greater frequency and severity of infections (4). Additionally, immune dysregulation from obesity is involved in the development of conditions such as metabolic syndrome and cardiovascular disease, which has been linked to immune activation even in the absence of infection (5). Thus, obesity in linked paradoxically with impaired immune responses yet elevated basal immune activation.

T cells, which are an integral part of the adaptive immune system, can be further divided into a range of T cell subsets that perform a diverse array of functions. These cells respond to the obese microenvironment such that obesity exhibits an alteration in the proportions and functions of certain T cells, such as an increase in the ratio of cluster of differentiation (CD)8$^+$ cytotoxic (Tc) cells to CD4$^+$ T helper (Th) cells (2). Within the CD4$^+$ Th cell population, there is an increase in the proportion of Th1 and Th17 cells, both of which function to increase the activation of other immune cells to a real or perceived threat (2). Furthermore, Th2 cells, which regulate immunologic memory, are decreased, as are T regulatory (Treg) cells, which suppress the immune response and maintain self-tolerance to prevent auto-immunity (2). Thus, this imbalance in T cell subsets with obesity creates an environment which promotes a non-specific inflammatory response, prevents generation of a robust adaptive immune response (6), and an environment which facilitates auto-immunity due to reduced self-tolerance (7).

While the immunologic consequences of obesity are well-studied, the factors driving this skewing of T cell differentiation are less clear. Obesity is accompanied by derangements in metabolic homeostasis such as metabolic inflexibility and insulin resistance (8, 9). Interestingly, metabolism directly impacts T cell differentiation and function by epigenetic modification and activation or silencing of key intracellular signalling pathways (6, 10). Co-engagement of the T cell receptor (TCR) and CD28 induces an upregulation of glycolysis via Akt signalling and glucose transporter (GLUT)1 expression in order to support cell activation and its greater metabolic demands (10). Following resolution of an infection, return to a quiescent state is required for formation of CD8$^+$ memory T cells. This relies on upregulated fat oxidation via activation of the nutrient sensors AMPK and inhibition of its downstream target mammalian target of rapamycin (mTORC)1, and increased expression of the fatty acid transporter carnitine palmitoyl transferase (CPT)-1 induces increased generation of CD8$^+$ memory T cells (10, 11). Contrary to effector T cell such as Th1 and Th17 cells, inhibition of mTORC1 and activation of AMPK facilitates differentiation of Treg cells which can occur in the absence of GLUT1 (11). Upregulation of fat oxidation by blocking glycolysis results in a preferential differentiation of Treg cells, however this does not restore proliferation of other effector T cells. Thus, an environment which favours glycolysis over fat oxidation may polarize T cells towards an activated state, while increased fat oxidation encourages pathogen memory and suppression of the inflammatory response (11).

Accordingly, manipulating metabolism to favour fat oxidation by fasting or intermittent fasting alters T cells ratios and activation. Reductions in the number and percent of circulating CD4$^+$ Th cells, as well as CD4$^+$ Th cell activation has been found to occur within as little as 24 hours of fasting in healthy humans (12) and longer duration studies (7-10 days) in humans have reported similar effects (13, 14). These changes in cell numbers have been found to occur due to a redistribution of CD4$^+$ Th cells to the bone marrow (15, 16) rather than apoptosis (17, 18). Mechanistically, fasting has been shown to increase Treg cells via COX2 (19), FOXO4 activation with fasting blunt Th1 and Th17 cytokine production (12), and fasting-induced suppression of leptin favours Th2 over Th1 differentiation (20). Thus, fasting appears to promote

a shift in T cell metabolism that may counteract T cell overaction in obesity, skewing T cell distribution towards memory forming and anti-inflammatory phenotypes. **Despite this, T cell metabolism during fasting has not yet been directly assessed. Additionally, whether fasting has the same effect on the differentiation and function of T cells from individuals with obesity as compared to lean individuals is still untested. Therefore, this study will help to provide insight into the therapeutic mechanisms by which fasting may improve immune dysregulation in obesity.**

METHODS

Recruitment and Eligibility

This two-group pre-post study will be registered on ClinicalTrials.gov once approval from the UBC Clinical Research Ethics Board has been granted. Participants will be recruited from the community and the UBC Okanagan campus with posters and social media posts. Additionally, an email invitation will be sent to participants from past studies that have indicated on their previous Consent Forms that they agree to be contacted for future research studies. We will aim to recruit equal numbers of males and females in each group.

Interested volunteers will be initially screened via a standardized phone call to determine if they meet the eligibility criteria. If the eligibility criteria are met, they will be invited to participate in the study, researchers will discuss the study details with the potential participant, and participants will be sent the Consent Form to review and sign if they consent to participate. This study will compare the responses of individuals with obesity to individuals that are lean. Lean individuals will be included if they have a body mass index (BMI) between 18.5 and 24.9 kg/m^2 and a waist circumference < 88 cm (females) or < 102 cm (males). Individuals with obesity will be included if they have a BMI of ≥ 30 kg/m^2 or a waist circumference ≥ 88 cm (females) or ≥ 102 cm (males). Lean individuals will be age- and sex-matched to individuals with obesity, and individuals in both groups will be included if they are 19-69 years of age and are physically inactive. This will be defined as accumulating < 150 minutes of moderate-to-vigorous physical activity per week or participating in moderate-to-vigorous physical activity < 3 days per week. Additionally, all participants must be able to read and understand English in order to complete the study diet logs. Interested volunteers will be excluded if they have a diagnosed autoimmune or inflammatory disease, have had a cancer diagnosis and/or treatment within the last 5 years, diagnosed type 1 or 2 diabetes, or a history of cardiovascular events (i.e., heart attack, stroke), and/or are currently pregnant. Additionally, interested volunteers will be excluded if they take glucose-lowering or thyroid medications, or if they currently smoke cigarettes or cannot refrain from smoking/using cannabis for the duration of the study. Finally, individuals that actively take ketone supplements, practice intermittent fasting with regular periods of fasting ≥ 24 hours, follow a ketogenic diet, or are actively trying to lose or gain weight (> 4 kg weight loss or gain in last month) will be excluded from the study.

Visits Summary

This study will involve a total of 3 visits across 48 hours during which participants will be in the laboratory for a total of 6 hours (study design pictured in **Figure 1**). Participants will be provided with a standardized meal at Visit 1, prior to beginning their fast. They will then fast for 48 hours, following which they will be provided with a standardized meal again at Visit 3, their final visit.

Once participants arrive at the lab for their first visit, we will obtain informed consent. Prior to arriving at the lab for their first visit, participants will have logged all of the food and drinks that they consume in a diary for the 3 days. Participants will bring this 3-day food log with them to their first visit to the lab. Additionally, we will ask participants not to consume any alcohol or participate in any exercise the day before their first visit.

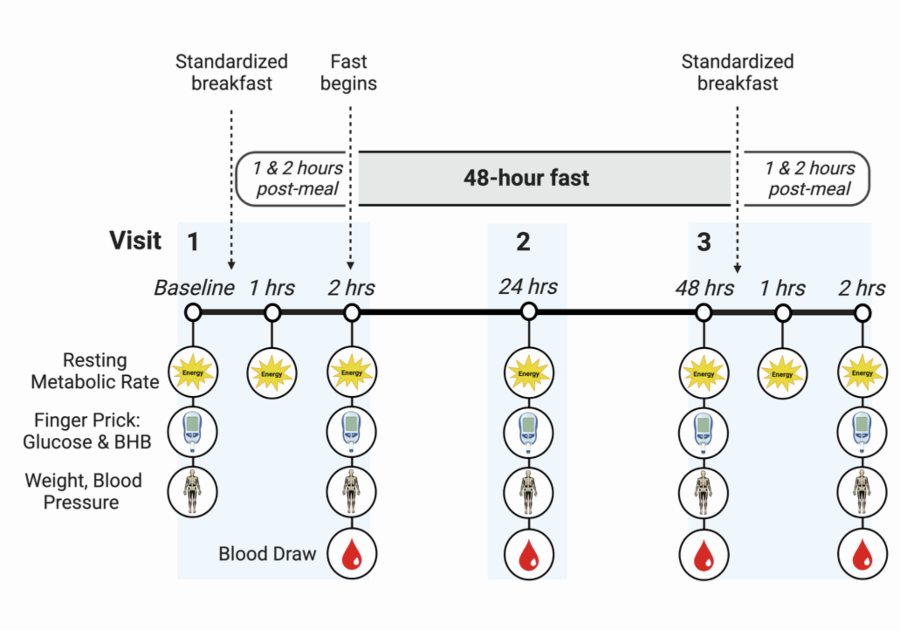

Visit 1: 2.5 hours

On the first visit, participants will arrive at the laboratory having fasted overnight for at least 10 hours. Once they arrive at the laboratory, researchers will measure their height, body weight, and blood pressure. Researchers will then measure participants' blood glucose and ketones by finger prick. Next, researchers will measure participants' resting metabolic rate (RMR; how much energy participants burn at rest). To do this, participants will rest lying down in a supine position and connected to a metabolic cart (Parvo Medics TrueOne 2400) with which their expired air will be collected and analyzed for 30 minutes. Once this is complete, participants will consume a standardized breakfast (meal replacement drink) and RMR will be measured 1 hour later. At 2 hours later, researchers will measure blood glucose and ketones again by finger prick, collect a venous blood sample (30 ml) into EDTA vacutainers, and measure RMR a final time. All blood samples will be obtained from the antecubital vein by a trained phlebotomist (Hashim Islam, PhD; Helena Neudorf, PhD student) using a standard 21-gauge needle. Approximately 30 ml of blood will be drawn into EDTA and sodium heparin vacutainers. Following this, participants' fast will begin. Participants will be instructed not to consume any food or beverages other than water or calorie-, sugar-, and caffeine-free beverages if they wish, or partake in any moderate-to-vigorous physical activity for the duration of their fast.

Visit 2: 45 minutes

Participants will arrive for their second visit 24 hours after the venous blood collection at the first visit (i.e., 26 hours after the beginning of participants' first visit). During this visit, 10 ml of blood will be obtained by a trained phlebotomist in the same manner as the first visit, and researchers will measure RMR again.

Visit 3: 2.5 hours

Participants will arrive for their third and final visit having fasted for 48 hours (i.e., 24 hours after their second visit). During this visit, researchers will measure weight and blood pressure, blood glucose and ketones by finger prick, collect a venous blood sample (30 ml) will be collected, and RMR will be measured again. Participants will then end their fast by consuming the same standardized breakfast drink to end their fast. Participants will remain in the laboratory for the next 2 hours, during which researchers will collect RMR 1 and 2 hours following participants' breakfast. At the end of the 2 hours, we will collect a final blood sample (10 ml) and measure blood glucose and ketones by finger prick a final time.

Statistical Analyses

Changes in all variables over time will be compared between the two groups using a linear mixed effects model with subject included as a random factor and group, time, and baseline measures will be included as fixed factors.

Sample Size

Previous studies examining differences in T cell numbers, function, and phenotype have reported medium to large effect sies effect sizes (d = 0.6-0.7) when comparing lean individuals and individuals with obesity (21, 22). Differences in resting whole body metabolism (e.g., metabolic flexibility, insulin resistance) between lean and obesity also typically yield medium to large effect sizes (d >1) (23–25). Therefore, we anticipate a medium-to-large effect size for the primary comparison between lean and obesity for T cell metabolism markers, counts, and function. Using an effect size of d=1.0, a two-tailed alpha of 0.05 and 80% power, N = 17 per group are estimated for an independent sample t-test (G*Power v3.1; output 1 below). This sample size is feasible for our study and also provides 86% power to detect a significant interaction (alpha 0.05) for a medium effect size (f = 0.25) for the 2 group X 2 timepoint design in the 48-fasting intervention, assuming a repeated measures correlation of r = 0.8 (output 2 below). A medium effect size is anticipated here based on the medium-to-large effect size seen in studies showing that fasting impacts T cell numbers (d = 0.8-1.0), using means and standard deviations and a repeated measures correlation of r = 0.8 (13, 14).

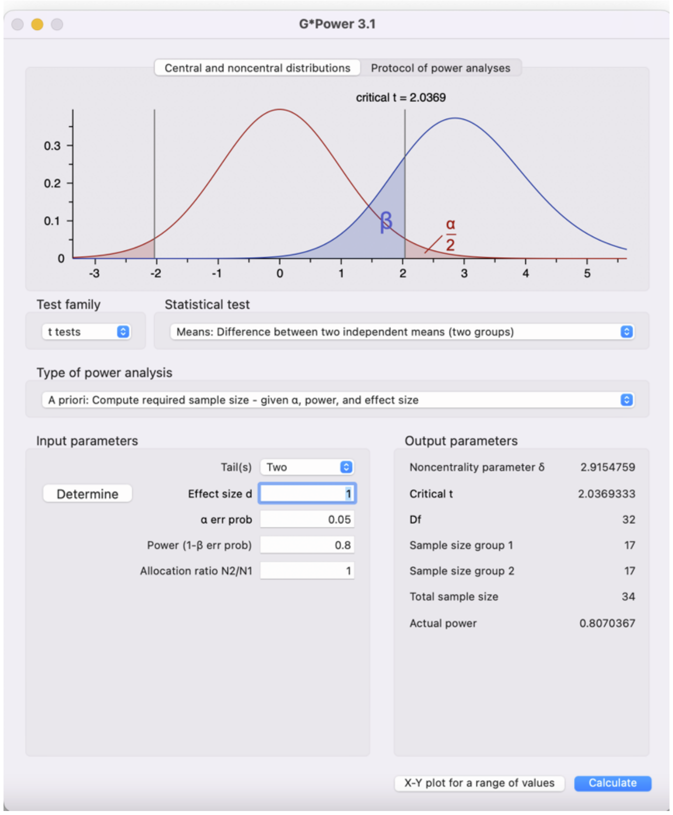

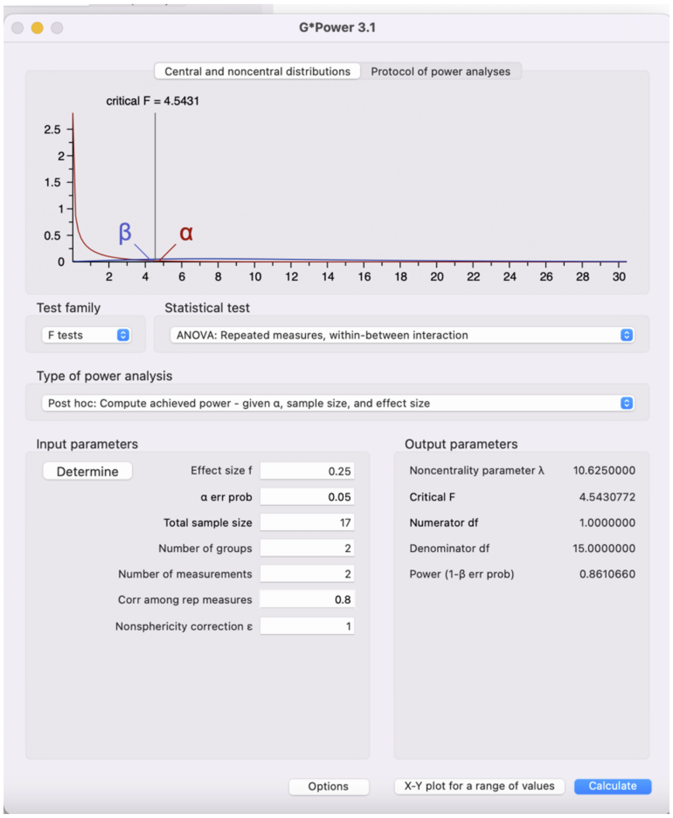

Significance

The results of this study will help to understand if T cell mitochondrial bioenergetics and metabolism differs between lean and obesity, while also determining if short-term fasting impacts the immune system differently in individuals with obesity compared to lean individuals. These data will help to inform future therapeutic applications of fasting or fasting-mimicking diets.

OUTCOMES MEASUREMENT AND METHODS

Anthropometric Measures

Height (cm), weight (kg), and waist circumference (cm) will be measured using standard procedures. Body mass index (BMI) will be calculated as kg/m2.

RMR

Resting metabolic rate (RMR) will be quantified by indirect calorimetry at the beginning of the first and second visit, and once every hour for 3 hours at the third and final visit. To do this, participants will be fitted with a mask and rest lying in a supine position for 30 minutes. Their expired air will be collected via a hose connected to the mask which will be analyzed by a metabolic cart (Parvo Medics TrueOne 2400).

Blood Sampling

Venous blood samples for quantifying T cell metabolism, subsets, and function will be obtained by a researcher trained in phlebotomy (Dr. Hashim Islam, PhD; Helena Neudorf, PhD Student). The procedure will follow sterile conditions within our laboratory (ART 115) with biosafety and

blood collection accreditation as approved previously (e.g., H21-01762, H21-03417). Blood will be collected into vacutainer tubes and immediately processed during all 3 visits. On the first visit, 30 ml of blood will be collected, 20 ml of blood on the second, 30 ml of blood at the end of the 48-hour fast, and 20 ml at the end of the 3-hour postprandial period.

First, pan T cells will be immediately isolated from 22 ml EDTA blood using an EasySep Direct Human T Cell Isolation Kit (StemCell Technologies; Cat No. 19661). T cells will be assessed in the basal and activated state. Basal cells will be assessed immediately. To activate T cells, T cells will be diluted in autologous media (RPMI 1640; 10 % autologous serum obtained from PBMC isolation; 1 % penicillin and streptomycin) and divided into 2 aliquots each containing 8 x 10^7 cells. These will be activated with anti-CD3 (1 ug/ml, ThermoFisher) and anti-CD28 (5 ug/ml, ThermoFisher) for 24 hours and then harvested. To quantify T cell metabolism, isolated pan T cells will be permeabilized using digitonin followed by the addition of complex I (CI; pyruvate, malate, glutamate) and II (CII; succinate) linked substrates in the absence or presence of ADP to determine oxygen consumption due to oxidative phosphorylation. Fat supported respiration (CI+II) will be subsequently assessed via addition of palmitoyl carnitine. Mitochondrial membrane integrity will be assessed using the addition of cytochrome c. Next, respiration due to protein leak will be determined following the addition of the ATP synthase inhibitor oligomycin. Maximal capacity of the electron transport system (ETS) will be determined by adding the uncoupler, CCCP (reflecting max ETS capacity through CI+CII), followed by CI inhibition using rotenone (reflecting max ETS through CII). To account for non-mitochondrial oxygen consumption, antimycin A will be used to inhibit CIII and this value will be subtracted from all other parameters. Finally, TPMD will be used to drive CIV activity through cytochrome c reduction providing an index of maximal CIV activity.

Peripheral blood mononuclear cells will be collected from 3 ml heparinized blood. To quantify T regulatory (Treg) suppressor function, PBMCs will be counted and diluted to 1-3 x 10^6 cells/ml in autologous media (RPMI 1640; 10 % autologous serum obtained from PBMC isolation; 1 % penicillin and streptomycin). Diluted PBMCs will be aliquoted into 4 wells (1.5 ml) in a 6-well plate with or without anti-CD3 (1 ug/ml, ThermoFisher) and anti-CD28 (5 ug/ml, ThermoFisher) or the FOXP3 inhibitor P60 (100 μmol, Abbiotec). PBMCs will be cultured in a 5% CO_2 incubator at 37°C for 24 hours. PBMCs will then be harvested and stained for the T cell markers CD3, CD4, CD8 and the activation markers CD25, CD71, and CD154 for 15 minutes in the dark at room temperature. Cells will be washed twice and analyzed immediately on the flow cytometer (Beckman Coulter).

To quantify T cell subsets, 500ul of PBMCs (1-3 x 10^6 cells/ml) diluted in autologous media (RPMI 1640; 10 % autologous serum obtained from PBMC isolation; 1 % penicillin and streptomycin; 1x Brefeldin A, ThermoFisher) will be collected and stimulated for 6 hours with anti-CD3 (1 ug/ml, ThermoFisher) and anti-CD28 (5 ug/ml, ThermoFisher) in a 5% CO_2 incubator at 37°C. PBMCs will be collected and washed twice in PBS and divided into two 200ul aliquots. The first aliquot will be stained to discriminate T effector cell subsets using the surface markers CD3, CD4, CD8, and CCR10 (26). The second aliquot will be stained to discriminate Treg cells using the surface marker CD3, CD4, CD25, and CD127 (27). Following incubation with surface stains for 15 minutes in the dark at room temperature, cells will be fixed (Fixation Reagent A, ThermoFisher) and incubated for 15 minutes at room temperature in the dark. Following two washes in PBS, cells will be permeabilized (Permeabilization Reagent B, ThermoFisher) and stained with intracellular stains for 20 minutes at room temperature in the dark. The intracellular stains for the T effector cell subsets will be IFNγ and IL-4 and the intracellular stain for the T regulatory cell panel will be FOXP3. Samples will be washed twice in PBS and immediately analyzed by flow cytometry (Beckman Coulter). T helper (Th) and T cytotoxic (Tc) cells will be identified by $CD3^+/CD4^+$ or $CD3^+/CD8^+$ staining. These populations'

subsets will be further discriminated as follows: Th/c1: IFNy^{+}/IL-4^{-}; Th2: IFNy^{-}/IL-4^{+}; Th/c17: IFNy^{-}/IL-4^{-}/IL-17^{+}; Th22: IL-4^{-}/IL-17^{-}/CCR10^{+}. Treg cells will be identified by CD3^{+}/CD4^{+}/CD25^{++}/CD127^{+}/FOXP3^{+} staining.

Standard metabolic (plasma glucose, free fatty acids, ketones, and insulin) and inflammatory (complete blood count, cytokines) will also be assessed as we have described previously (28, 29).

PROTECTION OF HUMAN PARTICIPANTS

Safety During Blood Sampling and Fasting

Blood Sampling: The insertion of a needle for blood sampling (intravenous and finger pricks) is a common medical practice and involves minimal risk provided proper precautions are taken. There may be some discomfort, discoloration/bruising with the cannula/venipuncture needle insertion; however, the risk of pain and infection will be minimized by using an experienced trained phlebotomist, and of course by ensuring the needle is inserted and removed under sterile conditions.

Fasting: Short-term fasting up to 72 hours is frequently used in research involving both lean healthy (30–32) and overweight/obese populations (8, 33–35) outside clinical/hospital settings to study the metabolic responses to fasting and underlying physiology with no reported adverse events. Researchers will stay in close contact with participants (check-in every 12 hours via phone or email) during their fast in case they experience any adverse events. Should participants feel faint or dizzy at any point during their fast they will be instructed to immediately end their fast. Additionally, we will have Dr. Kenneth Madden providing medical supervision for participants during their fast.

Protection of Personal Data

All participants will be given a unique study code, with all data and information gathered connected to this code. Only the PI will have access to the master list link the codes with participant names. Participant information and data will be either stored in a locked filing cabinet in the PI’s office, or on an internal UBC network drive accessed only through a dedicated LAN internet connection on a password protected computer in the PI’s laboratory with Salto pass card access only to the members of the research lab.

Informed Consent and Ethics

Prior to beginning the study, written informed consent will be obtained from participants and they will be informed that they can withdraw from the study at any time without consequence.

REFERENCES

1. Overweight and obese adults, 2018 [Online]. Statistics Canada. 2019-06-25 [6 Dec. 2022].

2. **Park C-S**, **Shastri N**. The Role of T Cells in Obesity-Associated Inflammation and Metabolic Disease. *Immune network* 22: e13–e13, 2022. doi: 10.4110/in.2022.22.e13.

3. **Andersen CJ**, **Murphy KE**, **Fernandez ML**. Impact of Obesity and Metabolic Syndrome on Immunity. *Advances in nutrition (Bethesda, Md)* 7: 66–75, 2016. doi: 10.3945/an.115.010207.

4. **Tagliabue C**, **Principi N**, **Giavoli C**, **Esposito S**. Obesity: impact of infections and response to vaccines. *European Journal of Clinical Microbiology and Infectious Diseases* 35: 325–331, 2016. doi: 10.1007/s10096-015-2558-8.

5. **Khafagy R**, **Dash S**. Obesity and Cardiovascular Disease: The Emerging Role of Inflammation. *Frontiers in cardiovascular medicine* 8: 768119–768119, 2021. doi: 10.3389/fcvm.2021.768119.

6. **Green WD**, **Beck MA**. Obesity altered T cell metabolism and the response to infection. *Current opinion in immunology* 46: 1–7, 2017. doi: 10.1016/j.coi.2017.03.008.

7. **Karczewski J**, **Zielińska A**, **Staszewski R**, **Eder P**, **Dobrowolska A**. Metabolic link between obesity and autoimmune diseases. *European cytokine network* 32: 64–72, 2021. doi: 10.1684/ecn.2021.0474.

8. **Bak AM**, **Møller AB**, **Vendelbo MH**, **Nielsen TS**, **Viggers R**, **Rungby J**, **Pedersen SB**, **Jørgensen JOL**, **Jessen N**, **Møller N**. Differential regulation of lipid and protein metabolism in obese vs. lean subjects before and after a 72-h fast. *American journal of physiology: endocrinology and metabolism* 311: E224–E235, 2016. doi: 10.1152/ajpendo.00464.2015.

9. **Wijngaarden MA**, **van der Zon GC**, **van Dijk KW**, **Pijl H**, **Guigas B**. Effects of prolonged fasting on AMPK signaling, gene expression, and mitochondrial respiratory chain content in skeletal muscle from lean and obese individuals. *American journal of physiology: endocrinology and metabolism* 304: E1012–E1021, 2013. doi: 10.1152/ajpendo.00008.2013.

10. **Bantug GR**, **Galluzzi L**, **Kroemer G**, **Hess C**. The spectrum of T cell metabolism in health and disease. *Nature reviews Immunology* 18: 19–34, 2018. doi: 10.1038/nri.2017.99.

11. **Newton R**, **Priyadharshini B**, **Turka LA**. Immunometabolism of regulatory T cells. *Nature immunology* 17: 618–625, 2016. doi: 10.1038/ni.3466.

12. **Han K**, **Singh K**, **Rodman MJ**, **Hassanzadeh S**, **Wu K**, **Nguyen A**, **Huffstutler RD**, **Seifuddin F**, **Dagur PK**, **Saxena A**, **McCoy JP**, **Chen J**, **Biancotto A**, **Stagliano KER**, **Teague HL**, **Mehta NN**, **Pirooznia M**, **Sack MN**. Fasting-induced FOXO4 blunts human CD4+ T helper cell responsiveness. *Nature metabolism* 3: 318–326, 2021. doi: 10.1038/s42255-021-00356-0.

13. **Komaki G**, **Kanazawa F**, **Sogawa H**, **Mine K**, **Tamai H**, **Okamura S**, **Kubo C**. Alterations in lymphocyte subsets and pituitary-adrenal gland-related hormones during fasting. *The American journal of clinical nutrition* 66: 147–52, 1997.

14. **Savendahl L**, **Underwood LE**. Decreased interleukin-2 production from cultured peripheral blood mononuclear cells in human acute starvation. *The Journal of clinical endocrinology and metabolism* 82: 1177–80, 1997.

15. **Shushimita S**, **Grefhorst A**, **Steenbergen J**, **de Bruin RWF**, **Ijzermans JNM**, **Themmen APN**, **Dor FJMF**. Protection against renal ischemia-reperfusion injury through hormesis? Dietary intervention versus cold exposure. *Life sciences* 144: 69–79, 2016. doi: 10.1016/j.lfs.2015.11.022.

16. **Shushimita S**, **De Jong - de Bruijn M**, **de Bruin R**, **IJzermans JNM**, **Heniks R**, **Dor F**. Dietary Restriction and Fasting Arrest B and T Cell Development and Increase Mature B

and T Cell Numbers in Bone Marrow. *PloS one* 9: e87772–e87772, 2014. doi: 10.1371/journal.pone.0087772.

17. **Takakuwa T**, **Nakashima Y**, **Koh H**, **Nakane T**, **Nakamae H**, **Hino M**. Short-Term Fasting Induces Cell Cycle Arrest in Immature Hematopoietic Cells and Increases the Number of Naive T Cells in the Bone Marrow of Mice. *Acta haematologica* 141: 189–198, 2019. doi: 10.1159/000496096.

18. **Mooren FC**, **Krueger K**, **Ringseis R**, **Eder K**, **Liebisch G**, **Conrad K**, **Alack K**, **Hajizadeh Maleki B**. Combined effects of moderate exercise and short-term fasting on markers of immune function in healthy human subjects. *American journal of physiology Regulatory, integrative and comparative physiology* 318: R1103–R1115, 2020. doi: 10.1152/ajpregu.00341.2019.

19. **Wang C**, **Zhang X**, **Luo L**, **Luo Y**, **Yang X**, **Ding X**, **Wang L**, **Le H**, **Feldman LER**, **Men X**, **Yan C**, **Huang W**, **Feng Y**, **Liu F**, **Yang XO**, **Liu M**. Adipocyte-derived PGE2 is required for intermittent fasting-induced Treg proliferation and improvement of insulin sensitivity. *JCI insight* 7, 2022. doi: 10.1172/jci.insight.153755.

20. **Saucillo DC**, **Gerriets VA**, **Sheng J**, **Rathmell JC**, **Maciver NJ**. Leptin metabolically licenses T cells for activation to link nutrition and immunity. *Journal of immunology (Baltimore, Md : 1950)* 192: 136–44, 2014. doi: 10.4049/jimmunol.1301158.

21. **NIEMAN DC**, **HENSON DA**, **NEHLSEN-CANNARELLA SL**, **EKKENS M**, **UTTER AC**, **BUTTERWORTH DE**, **FAGOAGA OR**. Influence of Obesity on Immune Function. *Journal of the American Dietetic Association* 99: 294–299, 1999. doi: 10.1016/S0002-8223(99)00077-2.

22. **Taylor JM**, **Li A**, **McLachlan CS**. Immune cell profile and immune-related gene expression of obese peripheral blood and liver tissue. *FEBS letters* 596: 199–210, 2022. doi: 10.1002/1873-3468.14248.

23. **Marzullo P**, **Verti B**, **Savia G**, **Walker GE**, **Guzzaloni G**, **Tagliaferri M**, **Di Blasio A**, **Liuzzi A**. The Relationship between Active Ghrelin Levels and Human Obesity Involves Alterations in Resting Energy Expenditure. *The journal of clinical endocrinology and metabolism* 89: 936–939, 2004. doi: 10.1210/jc.2003-031328.

24. **Verga S**, **Buscemi S**, **Caimi G**. Resting energy expenditure and body composition in morbidly obese, obese and control subjects. *Acta diabetologica* 31: 47–51, 1994. doi: 10.1007/BF00580761.

25. **Manzoni G**, **Oltolini A**, **Perra S**, **Muraca E**, **Ciardullo S**, **Pizzi M**, **Castoldi G**, **Lattuada G**, **Pizzi P**, **Perseghin G**. Resting Whole Body Energy Metabolism in Class 3 Obesity; from Preserved Insulin Sensitivity to Overt Type 2 Diabetes. *Diabetes, metabolic syndrome and obesity* 13: 489–497, 2020. doi: 10.2147/DMSO.S228229.

26. **Mousset CM**, **Hobo W**, **Woestenenk R**, **Preijers FW**, **Dolstra H**, **Waart AB van der**. Comprehensive Phenotyping of T Cells Using Flow Cytometry. *Cytometry Part A* 95: 647–654, 2019. doi: 10.1002/cyto.a.23724.

27. **Santegoets SJ**, **Dijkgraaf EM**, **Battaglia A**, **Beckhove P**, **Britten CM**, **Gallimore A**, **Godkin A**, **Gouttefangeas C**, **Gruijl TD de**, **Koenen HJ**, **Scheffold A**, **Shevach EM**, **Staats J**, **Taskén K**, **Whiteside TL**, **Kroep JR**, **Welters MJ**, **Burg SH van der**. Monitoring regulatory T cells in clinical samples: consensus on an essential marker set and gating strategy for regulatory T cell analysis by flow cytometry. *Cancer Immunology Immunotherapy* 64: 1271–1286, 2015. doi: 10.1007/s00262-015-1729-x.

28. **Walsh JJ**, **Neudorf H**, **Little JP**. 14-Day Ketone Supplementation Lowers Glucose and Improves Vascular Function in Obesity: A Randomized Crossover Trial. *The journal of clinical endocrinology and metabolism* 106: e1738–e1754, 2021. doi: 10.1210/clinem/dgaa925.

29. **Islam H**, **Jackson GS**, **Yoon JSJ**, **Cabral-Santos C**, **Lira FS**, **Mui AL**, **Little JP**. Sex differences in IL-10's anti-inflammatory function: greater STAT3 activation and stronger inhibition of TNF-α production in male blood leukocytes ex vivo. *American Journal of Physiology: Cell Physiology* 322: C1095–C1104, 2022. doi: 10.1152/ajpcell.00091.2022.

30. **Frank P**, **Katz A**, **Andersson E**, **Sahlin K**. Acute exercise reverses starvation-mediated insulin resistance in humans. *American journal of physiology: endocrinology and metabolism* 304: E436–E443, 2013. doi: 10.1152/ajpendo.00416.2012.

31. **Hoeks J**, **van Herpen NA**, **Mensink MR**, **Kornips CFP**, **Van Beurden D**, **Hesselink MKC**, **Schrauwen P**. Prolonged fasting identifies skeletal muscle mitochondrial dysfunction as consequence rather than cause of human insulin resistance. *Diabetes (New York, NY)* 59: 2117–2125, 2010. doi: 10.2337/db10-0519.

32. **Vendelbo MH**, **Clasen BFF**, **Treebak JT**, **Moller L**, **Krusenstjerna-Hafstrom T**, **Madsen M**, **Nielsen TS**, **Stodkilde-Jorgensen H**, **Pedersen SB**, **Jorgensen JOL**, **Goodyear LJ**, **Wojtaszewski JFP**, **Moller N**, **Jessen N**. Insulin resistance after a 72-h fast is associated with impaired AS160 phosphorylation and accumulation of lipid and glycogen in human skeletal muscle. *American Journal of Physiology - Endocrinology and Metabolism* 302: 190–200, 2012. doi: 10.1152/ajpendo.00207.2011.

33. **Bak AM**, **Vendelbo MH**, **Christensen B**, **Viggers R**, **Bibby BM**, **Rungby J**, **Jørgensen JOL**, **Møller N**, **Jessen N**. Prolonged fasting-induced metabolic signatures in human skeletal muscle of lean and obese men. *PloS one* 13: e0200817–e0200817, 2018. doi: 10.1371/journal.pone.0200817.

34. **Høgild ML**, **Gudiksen A**, **Pilegaard H**, **Stødkilde-Jørgensen H**, **Pedersen SB**, **Møller N**, **Jørgensen JOL**, **Jessen N**. Redundancy in regulation of lipid accumulation in skeletal muscle during prolonged fasting in obese men. *Physiological reports* 7: e14285-n/a, 2019. doi: 10.14814/phy2.14285.

35. **Pedersen MH**, **Svart MV**, **Lebeck J**, **Bidlingmaier M**, **Stødkilde-Jørgensen H**, **Pedersen SB**, **Møller N**, **Jessen N**, **Jørgensen JOL**. Substrate Metabolism and Insulin Sensitivity During Fasting in Obese Human Subjects: Impact of GH Blockade. *The journal of clinical endocrinology and metabolism* 102: 1340–1349, 2017. doi: 10.1210/jc.2016-3835.
